# Two H3K36 methyltransferases differentially associate with transcriptional activity and enrichment of facultative heterochromatin in rice blast fungus

**DOI:** 10.1007/s42994-023-00127-3

**Published:** 2023-12-18

**Authors:** Mengting Xu, Ziyue Sun, Huanbin Shi, Jiangnan Yue, Xiaohui Xiong, Zhongling Wu, Yanjun Kou, Zeng Tao

**Affiliations:** 1 Ministry of Agriculture Key Laboratory of Molecular Biology of Crop Pathogens and Insects, Institute of Biotechnology, Zhejiang University, Hangzhou, 310058 China; 2 State Key Lab of Rice Biology and Breeding, China National Rice Research Institute, Hangzhou, 310021 China

**Keywords:** Ash1, Facultative heterochromatin, H3K36me2/3, *Magnaporthe oryzae*, Set2, Transcriptional regulation

## Abstract

Di- and tri-methylation of lysine 36 on histone H3 (H3K36me2/3) is catalysed by histone methyltransferase Set2, which plays an essential role in transcriptional regulation. Although there is a single H3K36 methyltransferase in yeast and higher eukaryotes, two H3K36 methyltransferases, Ash1 and Set2, were present in many filamentous fungi. However, their roles in H3K36 methylation and transcriptional regulation remained unclear. Combined with methods of RNA-seq and ChIP-seq, we revealed that both Ash1 and Set2 are redundantly required for the full H3K36me2/3 activity in *Magnaporthe oryzae*, which causes the devastating worldwide rice blast disease. Ash1 and Set2 distinguish genomic H3K36me2/3-marked regions and are differentially associated with repressed and activated transcription, respectively. Furthermore, Ash1-catalysed H3K36me2 was co-localized with H3K27me3 at the chromatin, and Ash1 was required for the enrichment and transcriptional silencing of H3K27me3-occupied genes. With the different roles of Ash1 and Set2, in H3K36me2/3 enrichment and transcriptional regulation on the stress-responsive genes, they differentially respond to various stresses in *M. oryzae*. Overall, we reveal a novel mechanism by which two H3K36 methyltransferases catalyze H3K36me2/3 that differentially associate with transcriptional activities and contribute to enrichment of facultative heterochromatin in eukaryotes.

**Supplementary Information:**

The online version contains supplementary material available at 10.1007/s42994-023-00127-3.

## Introduction

In eukaryotes, the fundamental repeating unit of chromatin is the nucleosome, formed by 147 bp DNA wrapped around a histone octamer containing two copies of histone H2A, H2B, H3 and H4 (Luger et al. 1997; Wang et al. 2018). The chromatin state of a target locus influences the accessibility of transcriptional machinery and is closely associated with its transcriptional activity (Michalak et al. 2019). In chromatin-based transcriptional regulation, histone modifications play a central role in modulating downstream events (Friedrich et al. 2019; Jambhekar et al. 2019). Histone methylation frequently occurs on the lysine (K) or arginine (R) residues of H3 and H4 histones, which is catalysed by histone methyltransferases and removed by histone demethylases. Histone lysine methylation can take the form of mono-, di-, and tri-methylation and sites can occur at H3K4, K9, K36, K79 and H4K20 (Michalak et al. 2019). The association of histone methylation with transcriptional activation or repression depends on modifying lysine sites and the number of methyl groups added. For example, histone H3K9me and H3K27me are usually hallmarks of repressed heterochromatin, whereas H3K4me and H3K36me are associated with actively transcribed euchromatin (Michalak et al. 2019; Zhang et al. 2015).

Set2, a member of the KMT3 family, serves as an RNA Polymerase II associated histone methyltransferase and catalyzes multiple methylation events (mono-, di-, tri-methylation) on the histone H3K36 residue (Molenaar and van Leeuwen 2022; Sharda and Humphrey 2022). The different methylations are distributed in a graded manner across coding regions with mono-methylation (H3K36me1) at the 5' ends, di-methylation (H3K36me2) in mid-coding regions and tri-methylation (H3K36me3) towards the 3' ends of genes (Molenaar and van Leeuwen 2022; Sharda and Humphrey 2022). The extent of methylation and its genomic distribution are determined by different factors that coordinate to achieve diverse functional outcomes. H3K36 methylation and Set2 methyltransferase are first studied and well described in budding yeast (Molenaar and van Leeuwen 2022; Sharda and Humphrey 2022). In many organisms, H3K36me is considered as an active marker on euchromatin, where it plays an essential role in transcriptional elongation, repression of cryptic transcription, alternative splicing, dosage compensation, DNA replication and DNA damage repair (Molenaar and van Leeuwen 2022; Pajoro et al. 2017; Venkatesh and Workman 2013).

In fungi, H3K36 methylation is also able to activate transcription and enriches in intergenic and intragenic regions of the genome (Freitag 2017; Lai et al. 2022; Venkatesh and Workman 2013). There is a single methyltransferase, Set2, to catalyze H3K36 methylation in yeast, whereas another H3K36 methyltransferase, Absent small or homeotic discs 1 (Ash1), is present in many filament fungi, such as *Neurospora crassa*,* Fusarium fujikuroi*, *Aspergillus flavus* and* Magnaporthe oryzae* (Bicocca et al. 2018; Janevska et al. 2018; Pham et al. 2015; Zhuang et al. 2022). Ash1 has a SET domain for histone methylation, which was initially characterized as Trithorax Group (TrxG) superfamily (Freitag 2017). In *N. crassa*, Set2 is responsible for catalyzing the majority of H3K36me3 to facilitate transcriptional activation, whereas Ash1 catalyzes H3K36me2 for transcriptional repression in the genome (Bicocca et al. 2018; Ferraro et al. 2021). Moreover, deletion of *SET2* and *ASH1* both results in abnormal fungal growth (Bicocca et al. 2018). In *F. fujikuroi*, Set2 is responsible for catalyzing H3K36 methylation in euchromatic regions, whereas Ash1 catalyzes H3K36 methylation in the sub-telomeric regions, including the accessory chromosome (Janevska et al. 2018). In *A. flavus*, AshA regulates H3K36me2, whereas SetB primarily catalyzes H3K36me3 in the nucleus, and both modifications are required for fungal virulence and mycotoxin production (Zhuang et al. 2022). Although these two H3K36 methyltransferases are present in these fungi, their roles in H3K36 methylation and transcriptional regulation are still unclear.

In *M. oryzae*, *SET2* and *ASH1* (*KMT2H*) are conserved in the genome and their deletions impair fungal growth and pathogenicity (Pham et al. 2015). However, the precise roles of Ash1 and Set2 in the H3K36 methylation and transcriptional regulation in *M. oryzae* have yet to be established. In this study, we established that Ash1 and Set2 are redundantly required for the full H3K36me2/3 activity in *M. oryzae*. Combined with the methods of RNA-seq and ChIP-seq, we show that Ash1 and Set2 distinguish genomic H3K36me2/3-marked regions in the chromatin, which are differentially associated with repressed and activated transcription, respectively. Furthermore, we observed that Ash1-catalysed H3K36me2 co-localized with H3K27me3 in the chromatin and Ash1 was also required for the enrichment and transcriptional silencing of H3K27me3-occupied genes. Finally, Ash1 and Set2 play different roles in responding to various stresses in *M. oryzae*, possibly resulting from their different contributions to H3K36me2/3 modification and transcriptional regulation of the target genes.

## Results

### Ash1 and Set2 are both required for fungal growth, development and pathogenicity in *M. oryzae*

In filamentous fungi, two H3K36 methyltransferases, Ash1 and Set2, are typically present in the genome. To identify orthologs in the *M. oryzae* genome, a BLASTp analysis was performed, resulting in the identification of two SET2-domain histone methyltransferases, namely Ash1 (MGG_02937, KMT2H) and Set2 (MGG_01661, KMT3), as depicted in Fig. S1. Ash1 has one major SET domain and two PostSET (cysteine-rich motif following a subdomain of SET domains), which lack AWS (associated with SET domains) and WW (domain with two Trp/W residues). However, Set2 possesses three domains, including the major SET domain, AWS and PostSET (Fig. S1). Although previous studies have reported that deletion of *ASH1* or *SET2* impaired fungal growth and pathogenicity (Pham et al. 2015), the role of Ash1 and Set2 in the H3K36 methylation and transcriptional regulation in *M. oryzae* remained to be elucidated.

**Fig. 1:**
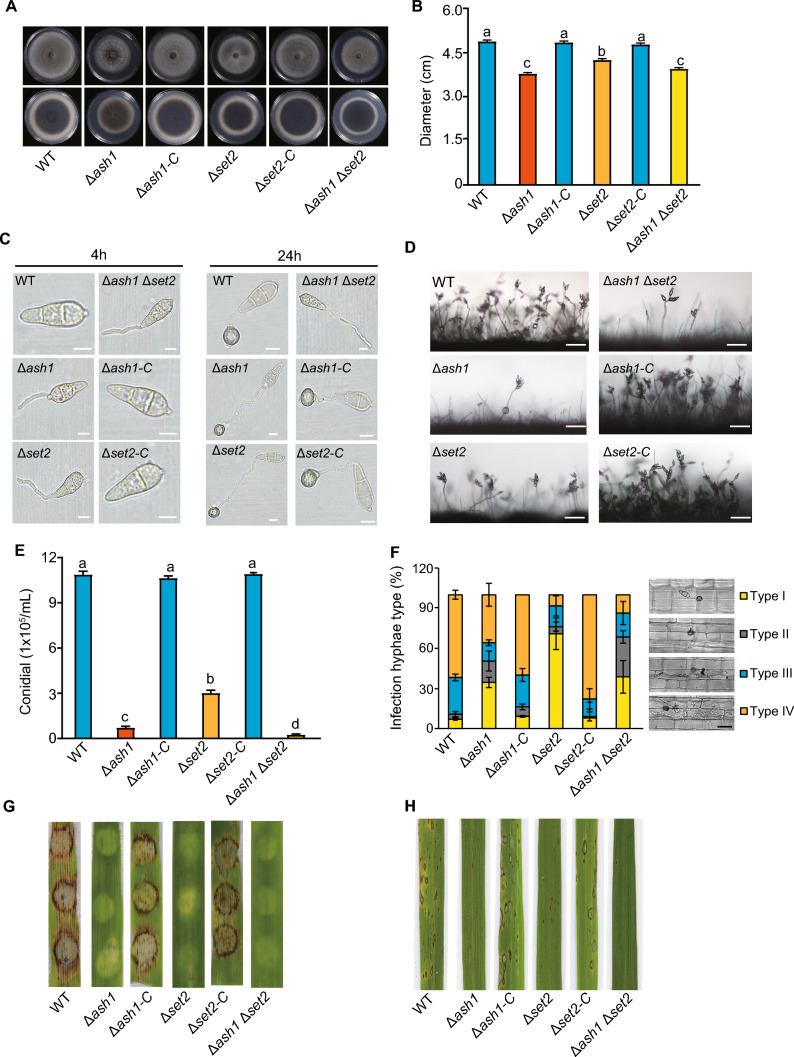
Ash1 and Set2 are required for fungal growth, conidiation and pathogenicity in *M. oryzae*. **A** Growth of wild-type (WT), deletion mutants and their complementary strains. Colonies of the indicated strains were grown on a complete medium (CM) for 7 d, then both the top and bottom sides of the colonies were imaged. **B** Statistical analysis of the colony diameters of the indicated strains grown on the CM. Values are the means ± SD from three biological repeats and different letters (a or b) indicate the significant differences, as determined by a one-way ANOVA (*P* < 0.05). **C** Conidia germination of the indicated strains. Strains were cultured in CM for 7 d, and the conidia were then collected to induce appressoria on a hydrophobic surface for 4 h and 24 h. Bar, 20 μm. **D** Conidiophore morphology of the indicated strains cultured in CM for 7 d. Bar, 200 μm. **E** Statistical analysis of conidiation of the indicated strains in CM. Values are means ± SD from three biological replicates. Different letters (a or b) indicate the significant differences, as determined by one-way ANOVA (*P* < 0.05). **F** Observation and statistical analysis of invasive hyphae growth in rice sheath cells at 40 hpi (hours post-inoculation). Four types of invasive hypha (illustrated in the right panel with the corresponding column) were quantified: no penetration, penetration with primary hyphae, penetration with secondary invasive hyphae in the first invaded cell, and invasive hyphae spreading into neighbouring cells. Data represent the mean ± SD of three independent repeats, with over 300 appressoria per analysis. Bar, 10 μm. **G**, **H** Infection assay with barely and rice seedlings. Susceptible barely and rice seedlings were used for infection and representative inoculated leaves are shown

To further establish the role of Ash1 and Set2 in *M. oryzae*, target deletion mutants of Δ*ash1* and Δ*set2*, as well as their double mutants, Δ*ash1*Δ*set2*, were developed in the WT (B157) strain (Fig. S2). Moreover, the complementary strains (Δ*ash1*-C and Δ*set2*-C) were generated in their deletion mutants, respectively. Subsequently, mycelia growth, conidia formation and pathogenicity were investigated in these deletion mutants and their complementary strains. When grown on complete medium (CM) for 7 d, both Δ*ash1* and Δ*set2* strains formed slightly smaller colonies compared with the WT strain (Fig. 1A and B). Although there were no significant changes observed in conidia germination and appressorium formation, between the deletion strains and WT, the number of conidia produced by Δ*ash1* and Δ*set2* was significantly reduced (Fig. 1C–E). Moreover, the proportion of type I infected mycelia in the deletion strains was significantly higher than that in the WT, thus impairing the ability to penetrate the host (Fig. 1F). Furthermore, the number of leaf spots infected by Δ*ash1*, Δ*set2* and Δ*ash1*Δ*set2* was significantly reduced through barley inoculation and rice spraying assays (Fig. 1G and H), which was accompanied by down-regulated expression of pathogenesis-related genes (Fig. S3). Together, deletion of *ASH1* and *SET2* significantly impaired fungal growth, development and pathogenicity in *M. oryzae*.

### Ash1 and Set2 are responsible for full H3K36me2/3 activity in *M. oryzae*

**Fig. 2:**
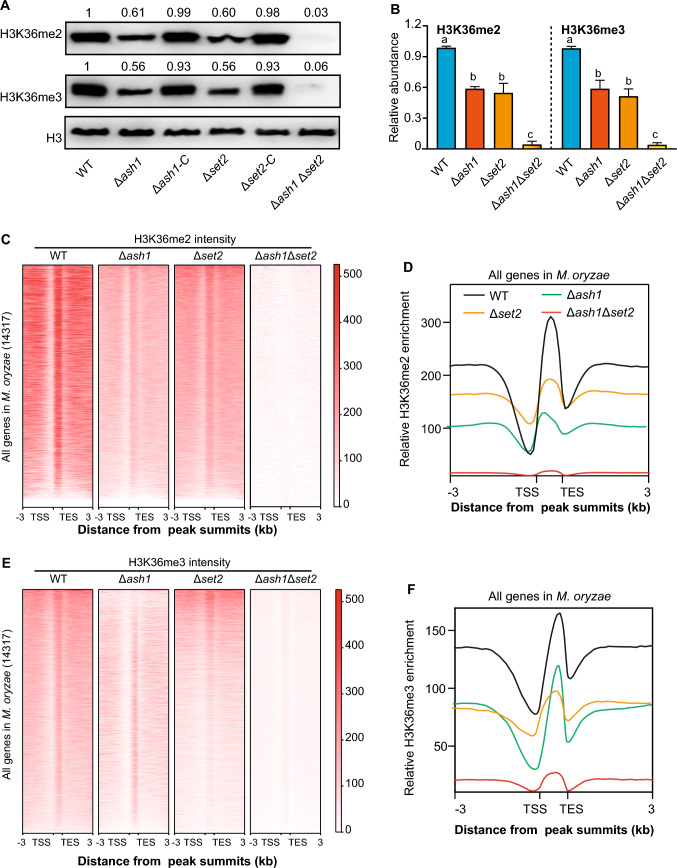
Ash1 and Set2 are responsible for the full activity of H3K36me2/3 in *M. oryzae.* **A** Western blotting assay in the WT, deletion mutants and their complementary strains. The relative intensity of H3K36me2 and H3K36me3 to H3 was calculated by ImageJ software, respectively, and WT was set as “1”. **B** The relative abundance of H3K36me2 and H3K36me3 in the WT and deletion mutants. Values are the means ± SD, from three biological replicates. Different letters (a or b) indicate significant differences, as tested by a one-way ANOVA (*P* < 0.05). **C** Heat maps showing occupancy of H3K36me2-established genes in the indicated strains. Transcriptional start site (TSS) and transcriptional termination site (TES) are shown. **D** Metagene plots showing the average H3K36me2 signal of all genes within 3.0-kb genomic regions flanking the peak summits in the WT and deletion strains. **E** Heat maps showing occupancy of H3K36me3-established genes in the indicated strains. **F** Metagene plots showing the average H3K36me3 signal of all genes within 3.0-kb genomic regions flanking the peak summits in the WT and deletion strains

We next assessed whether Ash1 and Set2 have H3K36 methyltransferase activity. First, subcellular localization of Ash1 and Set2 was investigated. A GFP-tagged *ASH1* or *SET2* fusion construct was transformed into an *H2B-mCherry* strain. As shown in Fig. S4, GFP-tagged Ash1 and Set2 fusions were co-localized with H2B-mCherry in the nucleus of mycelia and conidia. Subsequently, nuclear protein was extracted and relative abundance of H3K36me2 and H3K36me3 was detected with specific antibodies by Western blotting in the deletion mutants and their complementary strains. Notably, the abundance of H3K36me2 and H3K36me3 was almost absent in the Δ*ash1* Δ*set2* double mutant, while about half the amount was still retained in the single mutants Δ*ash1* or Δ*set2* (Fig. 2A and B, and Fig. S5). These results indicated that Ash1 and Set2 were redundantly responsible for full activity of H3K36me2/3 in *M. oryzae*.

Moreover, chromatin immunoprecipitation followed by high-throughput sequencing (ChIP-seq) was performed to map the genomic H3K36me2/3 occupancy in the Δ*ash1*, Δ*set2* and their double mutants. Compared with WT strain, the intensity of both H3K36me2 and H3K36me3 occupancy was barely detectable in the Δ*ash1* Δ*set2* deletion strain, which further indicated the role of Ash1 and Set2 as the histone methyltransferase of H3K36me2/3 (Fig. 2C–F). Compared with the WT strain, genomic regions with significant reduction of H3K36me2 and H3K36me3 occupancy in the Δ*ash1* and Δ*set2* strain were denoted as H3K36me2/3-marked peaks in *M. oryzae*. Collectively, 2061 significant H3K36me2 and 1478 H3K36me3 peaks (Log_2_FoldChange > 1, *P* < 0.05) were identified in the WT strain, which were associated with 1961 and 1452 genes, respectively. In addition, 462 genes were co-occupied with H3K36me2 and H3K36me3 (Fig. S6A). Together, these results further confirmed that Ash1 and Set2 were indispensably required for full H3K36me2/3 activity in *M. oryzae*.

### Ash1 and Set2 distinguish H3K36me2/3 occupancy, respectively

**Fig. 3:**
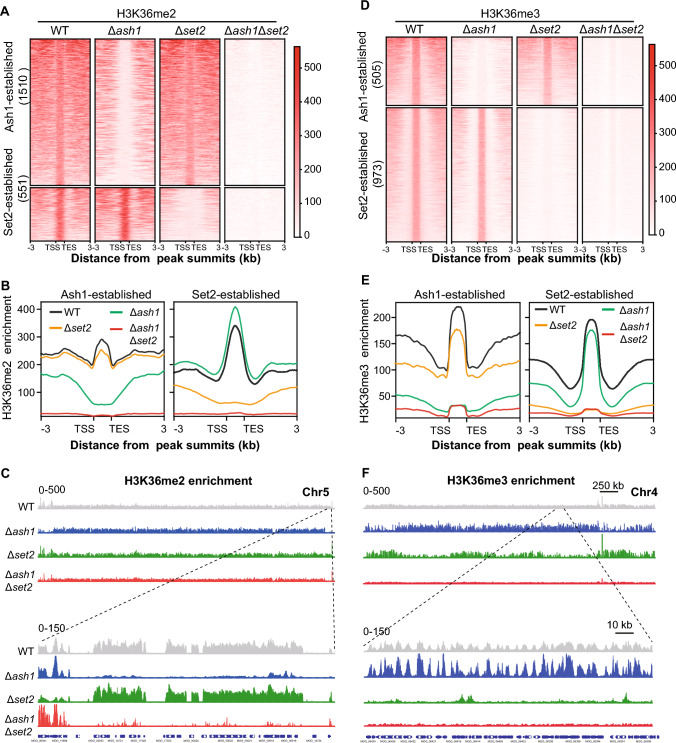
Ash1 and Set2 catalyze H3K36me2/3 in different genomic regions of *M. oryzae.* **A**–**C** Landscape, metagene plots and integrative genomics viewer of Ash1 and Set2-established H3K36me2 peaks in the indicated strains. The number of peaks is shown in the figure. Transcriptional start site (TSS) and transcriptional termination site (TES) are shown. **D**–**F** Landscape, metagene plots and integrative genomics viewer of Ash1 and Set2-established H3K36me3 peaks in the indicated strains

To further discover the individual roles of Ash1 and Set2 in the normal distribution of H3K36me2/3, the detailed occupancy catalyzed by Ash1 and Set2 was further investigated. Compared with the WT strain, genomic regions with a significant reduction of H3K36me2 and H3K36me3 occupancy in the Δ*ash1* strain were denoted as Ash1-established H3K36me2/3 peaks. Similarly, genomic regions with a significant reduction of H3K36me2/3 occupancy in the Δ*set2* strain were denoted as Set2-established H3K36me2/3 peaks. Overall, 1510 H3K36me2 peaks, associated with 1423 genes, were identified with a significant reduction in the Δ*ash1* strain, while 551 H3K36me2 peaks, associated with 540 genes, were identified in the Δ*set2* strain (Fig. 3A and B). In the H3K36me2-marked genes, nearly 72.6% (1423/1961) of genes were established by Ash1, and 27.5% (540/1961) were established by Set2. In contrast, only 34.2% (496/1452) of H3K36me3-marked genes were catalyzed by Ash1, whereas 65.8% (956/1452) genes were catalyzed by Set2 (Fig. 3C and D). Notably, there was almost no overlap of marked genes between Ash1-established H3K36me2 and H3K36me3, as well as almost no overlap between Set2-established H3K36me2 and H3K36me3, indicating the exclusive roles of Ash1 and Set2 in the same H3K36me2/3-catalyzed regions (Fig. S6B). By contrast, there was significant overlap of marked genes between Ash1-established H3K36me2 and Set2-established H3K36me3, as well as significant overlap between Set2-established H3K36me2 and Ash1-established H3K36me3 (Fig. S6B). Furthermore, genomic regions marked with H3K36me2 were mainly distributed in the promoter regions, while those marked with H3K36me3 were distributed in the exon regions (Fig. S6C). Together, these results established that Ash1 and Set2 have individual and interconnected roles in the genomic distribution of H3K36me2 and H3K36me3 in *M. oryzae*.

### Ash1 and Set2 differentially associate with repressed and activated transcription, respectively

**Fig. 4:**
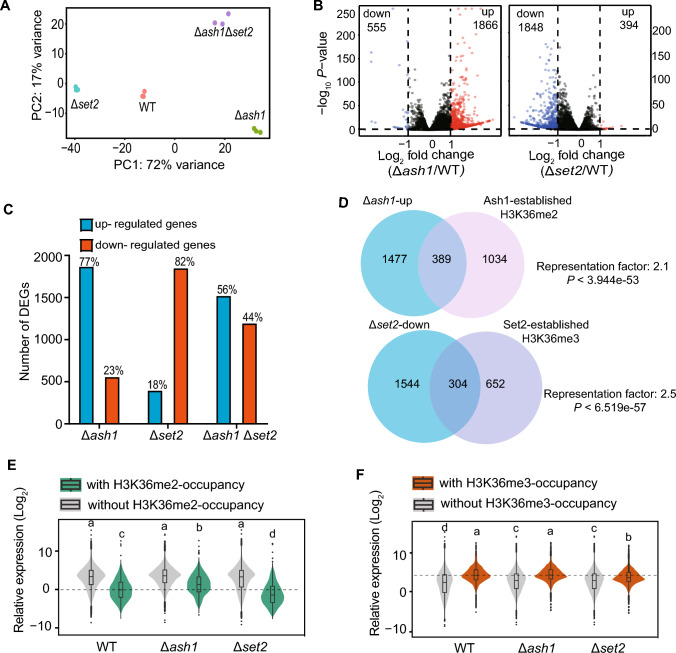
Ash1 and Set2 differentially associate with activated and repressed transcription. **A** Principal component analysis (PCA) showing differences in the transcriptomes of the indicated deletion strains compared with WT. Three biological repeats were performed for each strain in RNA-seq assays. **B** Volcano plot showing all differentially expressed genes (DEGs) of the indicated deletion strains compared with WT. **C** The number of up- and down-regulated genes in the indicated strains. The percentages are shown as the ratio of up- or down-regulated genes in all DEGs. **D** Venn diagram showing the significant overlap between up-regulated genes in *∆ash1* (*∆ash1*-up) and Ash1-H3K36me2-occupied genes, down-regulated genes in *∆set2* (*∆set2*-down) and Set2-H3K36me3-occupied genes, respectively. **E** Box plots and violin plots showing the average transcriptional levels of the genes with and without H3K36me2 occupancy in the indicated strains. **F** Box plots and violin plots showing the average transcriptional levels of the genes with and without H3K36me3 occupancy in the indicated strains

To explore whether Ash1 and Set2-catalyzed H3K36me2/3 occupancy associated with transcriptional expression, RNA-seq were performed in the WT, Δ*ash1*, Δ*set2* and their double mutants. Principal component analysis (PCA) revealed that the transcriptome between Δ*ash1* and Δ*set2* showed different directions compared with that of the WT strain, whereas the double mutants have relatively similar transcriptomes with deletion of *ASH1* (Fig. 4A). Collectively, deletion of *ASH1* resulted in 2421 differentially expressed genes (DEGs) compared with that in the WT strain, in which 1866 genes were up-regulated (Log_2_FC > 1, *P* < 0.05) and 555 genes were down-regulated (Log_2_FC < − 1, *P* < 0.05) (Fig. 4B). Loss of *SET2* caused 2242 DEGs, including 394 up-regulated genes and 1848 down-regulated genes (Fig. 4B). The absolute majority (77%, 1866/2421) of DEGs in the Δ*ash1* mutant were up-regulated genes, whereas the majority (82%, 1848/2242) in Δ*set2* were down-regulated genes (Fig. 4C). Together, these asymmetrical distribution of DEGs in the Δ*ash1* and Δ*set2* mutants implied that Ash1 and Set2 mainly function as transcriptional repressor and activator, respectively.

Next, sets of up-regulated genes in the Δ*ash1* strain (Δ*ash1*-up) and down-regulated genes in the Δ*set2* strain (Δ*set2*-down) were compared with sets of H3K36me2/3-marked genes. We found that Δ*ash1*-up had significant overlaps with H3K36me2-marked genes, particularly with part of those genes established by Ash1 (Fig. 4D and Fig. S7A–D). At the same time, down-regulated genes in the Δ*set2* strain had significant enrichment with H3K36me3 occupancy, specifically with part of those genes established by Set2 (Fig. 4D and Fig. S7A–D). Moreover, the average transcription of genes with or without H3K36me2/3-marked in the WT and deletion strains were compared. As shown in Fig. 4E, F, the average transcription of H3K36me2-marked genes had a relative lower level than those genes without H3K36me2 occupancy, whereas transcription of H3K36me3-marked genes had a higher level than those genes without H3K36me3 occupancy. Furthermore, a positive correlation between H3K36me3 signals and gene transcription, but negative correlation between H3K36me2 signals and gene transcription, was determined in the WT strain (Fig. S7E, F). Together, these results indicated that Ash1-established H3K36me2 occupancy was specifically associated with transcriptional repression, whereas Set2-established H3K36me3 was correlated with transcription activation in *M. oryzae*.

### Ash1-established H3K36me2 was specifically co-localized with H3K27me3

**Fig. 5:**
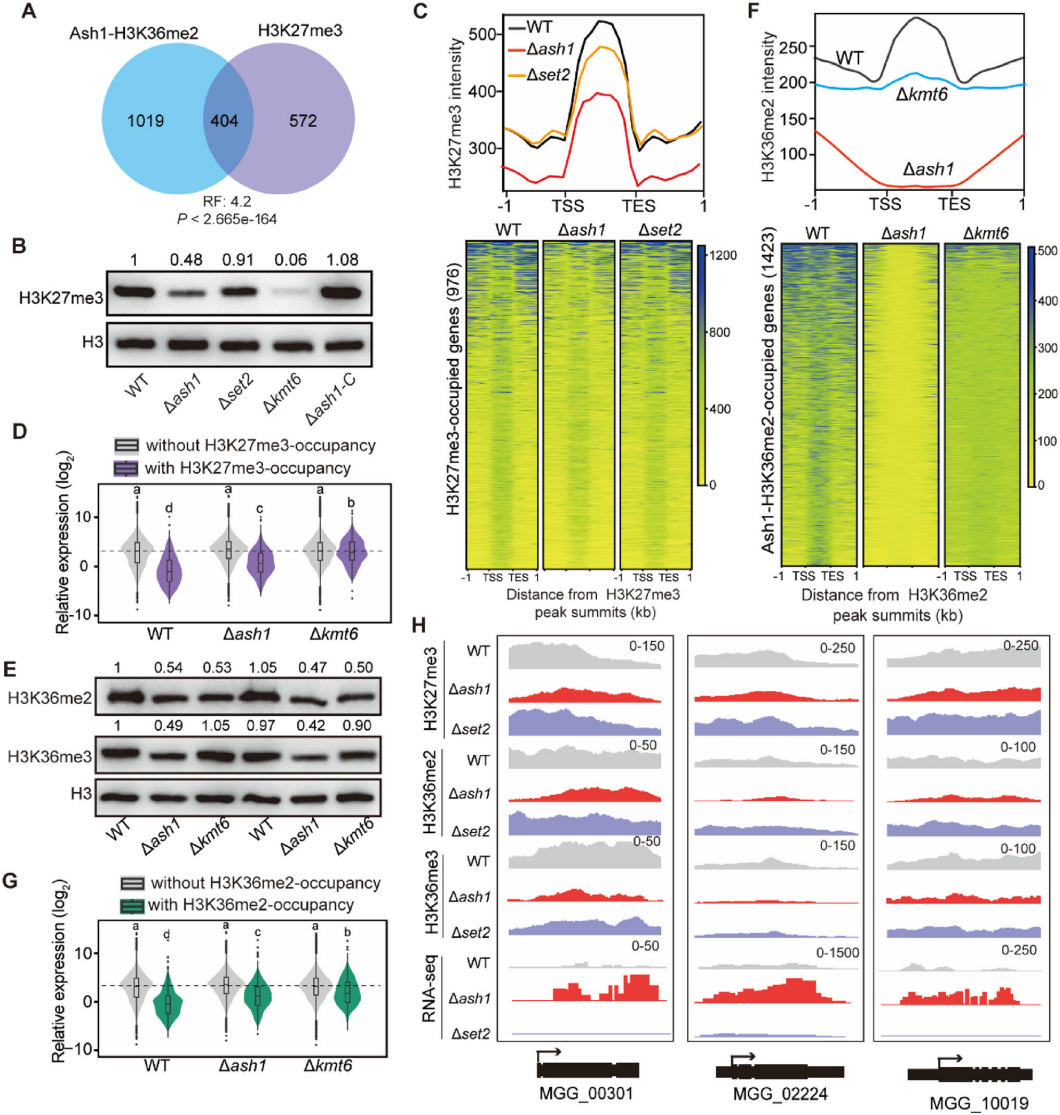
Ash1-H3K36me2 co-localizes with H3K27me3 occupancy in *M. oryzae.* **A** Venn diagram showing significant overlap between Ash1-H3K36me2 and H3K27me3-occupied genes. *RF* representation factor. **B** The relative abundance of H3K27me3 in the indicated strains. Ratio of H3K27me3 to H3 was calculated and the ratio in the WT strain was set at “1”. **C** Metagene plots and heatmap showing the average signal of H3K27me3 occupancy in the WT, *∆ash1* and *∆set2* strains. **D** Box plots and violin plots showing the average transcriptional levels of the genes, with and without H3K27me3 occupancy, in the indicated strains. **E** The relative abundance of H3K36me2 and H3K36me3 in the indicated strains. Ratio of H3K36me2/3 to H3 was calculated and the ratio in the WT strain was set at “1”. **F** Metagene plots and heatmap showing the average signal of H3K27me3 occupancy in the WT, *∆ash1* and *∆kmt6* strains. **G** Box plots and violin plots showing the average transcriptional levels of the genes with and without Ash1-H3K36me2 occupancy in the indicated strains. **H** Integrative Genomics Viewer (IGV) of ChIP-seq and RNA-seq in the WT, *∆ash1* and *∆set2* strains are shown. The number areas were reads per million (RPM)

In our previous studies, we investigated the molecular mechanism for normal distribution of H3K27me3, a hallmark of facultative heterochromatin in *M. oryzae* (Lin et al. 2022). Since Ash1-established H3K36me2 occupancy associates with transcriptional repression, we further explored whether Ash1-established H3K36me2 co-localized with, or affected, H3K27me3 occupancy. Gene sets of Ash1-established H3K36me2 occupancy and H3K27me3 occupancy in the WT strain were compared. As shown in Fig. 5A, 41.3% (404/976) of H3K27me3-marked genes were co-marked with H3K36me2 established by Ash1, but not Set2 (Fig. S8A). The specific co-localization of H3K27me3 and Ash1-established H3K36me2 implied that Ash1 contributes to H3K27me3-mediated transcriptional silencing.

Kmt6 is the core subunit that catalyzes H3K27me3 in the Polycomb repressive complex 2 (PRC2) in *M. oryzae* (Wu et al. 2022). We further compared the transcriptomes between Δ*ash1* and Δ*kmt6*. The DEGs between the Δ*ash1* and Δ*kmt6* strains exhibited the same direction of mis-regulation compared to the WT, sharing significant overlap in the upregulated genes (Fig. S8B). In addition, 50% (714 of 1423) of Ash1-H3K36me2-marked genes were de-repressed in the Δ*kmt6* strain and 21% (205 of 976) of H3K27me3-occupied genes were de-repressed in the Δ*ash1* strain (Fig. S8C-D). These results further indicated that Ash1-established H3K36me2 might coordinate with H3K27me3 to repress transcription in *M. oryzae*.

### Ash1 is required for the enrichment and transcriptional silencing of H3K27me3-occupied genes

We next assessed whether disruption of *ASH1* would affect H3K27me3 distribution. First, relative abundance of H3K27me3 was examined in the WT and deletion strains. As shown in Fig. 5B, H3K27me3 abundance was significantly reduced in Δ*ash1* and no obvious change in Δ*set2*, when compared with that in the WT and complementary strains (Fig. S9). Subsequently, genomic H3K27me3 occupancy in the WT, Δ*ash1* and Δ*set2* strains was further determined with ChIP-seq assay. Metaplot analysis indicated that the average signal of H3K27me3-occupied genes was significantly reduced in the Δ*ash1* strain, when compared with that in the WT and Δ*set2* strains (Fig. 5C). In addition, 175 H3K27me3 hypo-methylated and 162 hyper-methylated genes were identified in the Δ*ash1* strain compared those in the WT strain. Together, these results indicated that Ash1 is required for the enrichment of H3K27me3-occupied genes in *M. oryzae*.

Whether the co-localization of Ash1-established H3K36me2 with H3K27me3 and the changed enrichment of H3K27me3 in the Δ*ash1* strain would associate with transcriptional expression was further investigated. The average transcription of genes with or without H3K27me3-marked were compared. As shown in Fig. 5D, the average transcription of H3K27me3-marked genes in the Δ*ash1* strain had a higher level than those in the WT strain. These results indicated that Ash1 and Ash1-H3K36me2 were required for transcriptional silencing of H3K27me3-occupied genes.

We also investigated whether Kmt6 was required for the enrichment and transcriptional silencing of H3K36me2-occupied genes. As shown in Fig. 5E, abundance of H3K36me2, but not H3K36me3, was specially reduced in the Δ*kmt6* strain, which was further confirmed by ChIP-seq assays (Fig. 5F). Moreover, the average transcription of Ash1-H3K36me2-occupied genes in the Δ*kmt6* strain was higher than those in the WT strain (Fig. 5G). Therefore, Kmt6 is also necessary for the enrichment and transcriptional repression of Ash1-H3K36me2-occupied genes in *M. oryzae*.

### Ash1 and Set2 respond differentially to various stresses

As such differences in transcriptome and H3K36me2/3 occupancy were observed in the Δ*ash1* and Δ*set2* strains, gene ontology (GO) analysis was performed with gene sets of Δ*ash1*_up and Δ*set2*_down. Term of methylation, fatty acid and mycotoxin biosynthetic process, polysaccharide, mycotoxin and xylem catabolic process were significantly enriched in the Δ*ash1*, whereas term of transcription, carbohydrate metabolic process, transmembrane and carbohydrate transport, cellulose and polysaccharide catabolic process, cellular response to oxidative stress were significantly enriched in the Δ*set2* (Fig. S10). These findings implied that Ash1 and Set2 had different or unique biological functions in *M. oryzae*.

**Fig. 6:**
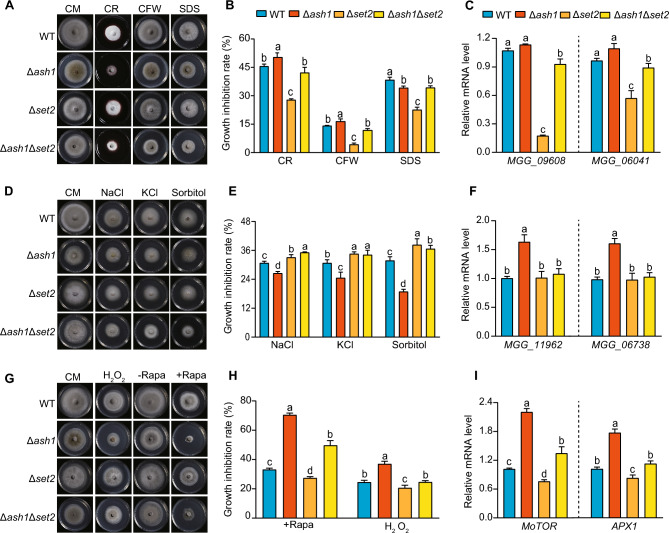
Ash1 and Set2 are differentially involved in response to various applied stresses. **A**, **B** Growth and statistical analysis of colony diameters of the indicated strains in CM, supplemented with CR, CFW and SDS. Values are the means ±  SD, with three biological replicates;  different letters (a or b) indicate significant differences, as tested by a one-way ANOVA (*P* < 0.05). **C** Relative expression levels of cell wall-related genes in the indicated strains. Values are the means ±  SD, with three biological replicates; different letter (a, b or c) indicate significant differences, as tested by a one-way ANOVA. **D**, **E** Growth and statistical analysis of colony diameters of the indicated strains in CM, supplemented with NaCl, KCl or Sorbitol. **F** Expression analysis of salt-related genes in the indicated strains. **G**, **H** Growth and statistical analysis of colony diameters of the indicated strains in CM, supplemented with oxidative stress and rapamycin (Rapa). **I** Expression analysis of *MoTOR* and *APX1* in the indicated strains

Subsequently, we further tested and compared whether Ash1 and Set2 differentially respond to various stresses. For cell wall stress, the sensitivity of the WT and deletion strains grown on the CM supplemented with sodium dodecyl sulphate (SDS), Congo red (CR), or calcofluor white (CFW) were tested. The growth inhibition was significantly reduced in the Δ*set2* strain, whereas no obvious change was detected in the Δ*ash1* strain, compared with that in the WT strain (Fig. 6A and B). Consistently, transcription of cell wall-related genes was specifically down-regulated in the Δ*set2* strain, accompanied by a changed H3K36me3 occupancy (Fig. 6C and Fig. S11). When strains were treated with salt stress, under KCl, NaCl or sorbitol, the growth inhibition was significantly reduced in the Δ*ash1* strain, whereas deletion of *SET2* had comparable sensitivity with that in the WT strain (Fig. 6D and E). Expression of stress-related genes was specifically up-regulated in the Δ*ash1* strain, accompanied by changed H3K36me2 occupancy (Fig. 6F and Fig. S11). Furthermore, deletion of *ASH1* or *SET2* also caused an opposite phenotype, when treatment with oxidative stress and rapamycin (Rapa) (Fig. 6G and H). Compared with WT, higher sensitivity was observed in the Δ*ash1* strain whereas higher tolerance was detected in the Δ*set2* strain (Fig. 6G and H). Expression of *MOTOR* and *APX1* has different patterns between the Δ*ash1* and Δ*set2* strains, which were up-regulated in the Δ*ash1* strain and down-regulated in the Δ*set2* strain (Fig. 6I and Fig. S11). However, the total abundance of H3K36me2 and H3K36me3, under the above stresses, such as SDS, NaCl and oxidative stress, had no obvious change between mock and stress treatments (Fig. S12). Together, these results further indicated that Ash1 and Set2 have different roles in response to various stresses, which might associate with their different roles in H3K36me2/3 establishment and transcriptional regulation in *M. oryzae*.

## Discussion

Although two conserved H3K36 methyltransferases, *ASH1* and *SET2*, have been identified in the genome, and their deletions impair fungal growth and pathogenicity in *M. oryzae* (Pham et al. 2015), it remained unclear whether and how they were involved in H3K36 methylation and transcriptional regulation. Here, we have elucidated the critical and distinct roles of Ash1 and Set2 in the di- and tri-methylation of H3K36 and transcriptional regulation. Moreover, our findings reveal that Ash1, but not Set2, is required for the distribution of facultative heterochromatic modifications and coordinately maintaining transcriptional silencing in *M. oryzae*.

H3K36me usually acts as an active marker on euchromatin and plays an essential role in transcriptional regulation (Freitag 2017; Lai et al. 2022). For instance, in the entomopathogenic fungus *Metarhizium robertsii*, Ash1-mediated H3K36me2 activates the peroxin gene *Mrpex16*, which is important for the biogenesis of peroxisomes that promotes appressorium turgor generation (Wang et al. 2023). *PsKMT3*, encoding H3K36 methyltransferases in *Phytophthora sojae*, is positively associated with gene expression (Chen et al. 2023). In our study, neither *ASH1* nor *SET2* is essential for fungal growth and development, which provided the possibility to assess their contributions to H3K36me2/3 activity. Ash1 is responsible for the majority of H3K36me2 catalyzation and Set2 for the majority of H3K36me3, which were differentially associated with transcriptional repression and activation, respectively. This redundancy and specific role of Ash1 and Set2 for proper H3K36me2/3 occupancy, at genes, may be beneficial to appropriate transcription under changed environments. Notably, some regions in the chromatin undergo di-methylation by one of these methyltransferases, only to be further tri-methylated by the other methyltransferase but not by itself. This finding also raised an interesting question of how to specifically “erase” Ash1-H3K36me2/3 and Set2-H3K36me2/3 in *M. oryzae*. Moreover, Ash1-H3K36me2 is associated with transcriptional repression in *N. crassa* and Ash1 catalyzes H3K36 methylation in the heterochromatic-liked regions, such as sub-telomeric regions and the accessory chromosome in *F. fujikuroi* (Bicocca et al. 2018; Janevska et al. 2018). In embryonic stem cells, H3K36me2 methyltransferase Nsd1 mediates H3K36me2 co-localization with PRC2-mediated H3K27me2 genome-wide (Streubel et al. 2018). Although Nsd1 modulates the activity of PRC2 to restrict H3K27me3 deposition, it could demarcate H3K27me3 from H3K27me2 domains (Streubel et al. 2018). In fission yeast, H3K36me2 is sufficient to recruit the Rpd3S histone deacetylase complex to repress cryptic transcription from transcribed regions (Suzuki et al. 2016). The different roles of H3K36me2 and H3K36me3 in catalytic and transcriptional activity would be necessary for the events based on their protein partners or tissue-specific expression patterns (Lam et al. 2022; Rajagopalan et al. 2021). Therefore, H3K36me cannot be universally considered as an active marker, especially in filamentous fungus.

The crosstalk of different histone methylations participates in the precise regulation of gene expression (Zhang et al. 2015). It is well known that Trithorax system acts, via methylation of histone H3K36 and H3K4, thereby inhibiting H3K27me activity of the Polycomb complexes (Piunti and Shilatifard 2021; Zhang et al. 2015). The trithorax group protein Kismet (KIS) promotes transcription elongation, facilitates the binding of the H3K36 methyltransferase Ash1 to active genes, and counteracts repressive methylation of H3K27 by Polycomb group proteins (Dorighi and Tamkun 2013). In *Drosophila*, Ash1 establishes H3K36 methylation to promote *HOX* gene expression by counteracting Polycomb silencing (Dorafshan et al. 2019; Miyazaki et al. 2013). In mammalian germ cells, NSD1-deposited H3K36me2 directs de novo methylation and counteracts Polycomb-associated silencing (Shirane et al. 2020). However, the results in filamentous fungus contrast with those published for yeast and higher eukaryotes. For example, Ash1-marked chromatin can be further modified with H3K27me in *N. crassa*, and Ash1 catalytic activity modulates the accumulation of H3K27me2/3 both positively and negatively (Bicocca et al. 2018; Ferraro et al. 2021). In our study, Ash1-catalyzed H3K36me2 associates with transcriptional silencing and is co-localized with H3K27me3. Moreover, Ash1 is required for the enrichment and transcriptional silencing of H3K27me3-occupied genes. So, we concluded that Set2-catalysed H3K36me3 similarly counteracts repressive H3K27me3 to activate transcription, whereas Ash1-catalyzed H3K36me2 may co-ordinate with H3K27me3 to repress transcription in *M. oryzae*.

Regulation on the establishment and maintenance of H3K27me3 occupancy remains poorly understood, especially in fungi (Ridenour et al. 2020; Wiles and Selker 2017). Polycomb proteins usually assemble to form polycomb repressive complex 1 (PRC1) and PRC2, of which PRC2 catalyzes H3K27me3 and PRC1 participates in H3K27me3 recruitment and contributes to chromatin compaction in higher plants and animals (Bieluszewski et al. 2021; Ridenour et al. 2020). Although PRC2 is highly conserved in fungi, no known subunits of PRC1 have been identified in the fungal genome so far. This raises questions about how PRC2 can efficiently perform transcriptional silencing in the absence of PRC1 (Ridenour et al. 2020; Wiles and Selker 2017). Accessory proteins of PRC2, H3K27me “reader” proteins and chromatin remodelling factors have been identified and are required for transcriptional silencing (Courtney et al. 2020; Ferraro et al. 2021; Jamieson et al. 2018; Kamei et al. 2021; Lin et al. 2022; Tang et al. 2021; Wiles et al. 2020, 2022). In our study, H3K27me3 occupancy co-localized with the repressive H3K36me2 and the average signal of H3K27me3 occupancy was reduced in the Δ*ash1* strain. Moreover, 50% of repressive H3K36me2-marked genes were upregulated in the Δ*kmt6* strain and 21% of H3K27me3-occupied genes were de-repressed in the Δ*ash1* strain. These findings shed light on a novel role and mechanism in the establishment and maintenance of facultative heterochromatin and stable maintenance of gene repression by H3K36 methyltransferase and its catalyzed H3K36me2 in *M. oryzae*. Further studies are required to investigate whether there is a direct, or indirect, physical connection between Ash1 and the Polycomb repressive complex. In embryonic stem cells, H3K36me2 methyltransferase Nsd1 was identified as a PRC2 associated protein and modulated PRC2 activity, establishing a direct connection for the two modifications (Streubel et al. 2018).

In conclusion, our research uncovers a novel mechanism for the opposite transcriptional activity by two histone methyltransferases, which catalyze H3K36me2 and H3K36me3, and their different roles in the distribution of facultative heterochromatin in eukaryotes. These new insights in distribution patterns and transcriptional effects of H3K36me2/3 broaden our current understanding of chromatin-based transcriptional regulation in eukaryotes.

## Materials and methods

### Fungal strains and culture conditions

*Magnaporth oryzae* strain B157 was used as the wild-type (WT) in this study for obtaining deletion mutants. Strains were grown on the complete medium (CM) at 25 °C for 7 d for growth measurement. For stress assessment, strains were grown on the CM supplemented with 10 mM H_2_O_2_, 50 µg/mL calcofluor white (CFW), 500 µg/mL Congo red (CR), 0.005% sodium dodecyl sulphate (SDS) and 200 ng/mL rapamycin (Macklin, R81729) at 25ºC in the darkness for 7 d. All experiments were conducted with three biological replicates and each replicate included at least three independent samples.

### Plasmid construction

To create the deletion mutants of *ASH1* and *SET2*, about 1.0-kb of 5’ untranslated region (UTR) and 3’ UTR was sequentially cloned to the flank of *resistance genes* cassette of *pFGL821* (Addgene, 58223) according to the standard one-step gene replacement strategy (Wu et al. 2022). To construct complementary plasmid of Δ*ash1-*C, a 6.0-kb genomic fragment containing 2.0-kb of upstream sequence and 3.3-kb coding sequence plus 0.7-kb 3' UTR was amplified and cloned into *pFGL823*. To create Δ*set2-*C, a 5.0-kb genomic fragment containing 2.0-kb of upstream sequence and 3.0-kb coding sequence fused with the *eGFP* and *TrpC* terminator was sequentially cloned to *pFGL820*. To generate *ASH1-GFP* and *SET2-GFP*, the coding regions of *ASH1* and *SET2*, derived with the *H3* promoter, were cloned to *pKD5-H3-GFP-SUR*. After confirmed by sequencing, the resulting plasmids were introduced into the WT or their deletion mutants with a method of *Agrobacterium tumefaciens*-mediated transformation (ATMT) (Wu et al. 2022). Strains and primers used in the experiments were listed in Table S1 and S2 respectively.

### Conidial morphology observation assay

For conidiation, strains were grown on CM at 25 °C in 16 h light /8 h dark for 7 d. The conidia were washed from the petri dish with ultra-clean water, followed by filtration and centrifugation. The number of spores was controlled with about 10^4^ mL^−1^ using a blood cell counting plate. Then, about 10 µL spore solution was carefully dripped and cultured on hydrophobic slides for 4 h and 24 h away from light. The remaining strain residues on the petri dish were cut off with sterile blades and placed on the carrier fragments for 24 h away from light. After that, the microscopic morphology of conidia and conidiophores was observed and photographed.

### Rice seedling and sheath infection assay

Rice seedling infections were conducted with a 5 × 10^4^ mL^−1^ conidia suspension. After 7 d infection, disease symptoms of infection assays were assessed with three repeats. For development assay of *in planta* invasive hyphae, 21-d-old healthy rice seedlings (CO39) were used for sheath preparation. A conidial suspension (5 × 10^4^ mL^−1^) was inoculated into the rice sheath, subsequently incubated in a growth chamber with a photoperiod of 16 h light and 8 h dark at 25 °C. The inoculated sheaths were trimmed manually and observed with an Olympus wide field microscope at 40 hpi.

### Subcellular localization

*H2B-mCherry* construct was described previously (Wu et al. 2023). *H2B-mCherry* was transformed and co-expressed in the *ASH1-GFP* and *SET2-GFP* strains respectively. Subcellular localization of Ash1-GFP, Set2-GFP and H2B-mCherry was observed in both the mycelia and conidia stages. The fluorescent signals of GFP and mCherry fusion were captured by a confocal fluorescence microscope (Zeiss LSM700) with 488 nm and 555 nm laser excitation respectively.

### Western blotting

0.5-g mycelia cultured in the liquid CM for 2 d were collected. The nuclei of mycelia were isolated with extraction buffer (20 mM Tris–HCl pH 7.5, 20 mM KCl, 2 mM MgCl_2_, 25% glycerol, 250 mM sucrose, 0.1 mM phenylmethylsulfonyl fluoride, 5 mM beta-mercaptoethanol, and 1 × cocktail proteinase inhibitor) and filtered with two layers of Miracloth (Millipore, 475855-1R). Subsequently, total histones were extracted with lysis buffer (50 mM Tris–HCl pH 7.4, 150 mM NaCl, 1 mM EDTA, 1% Triton X-100 and 1 × cocktail proteinase inhibitor) and separated by 15% SDS-PAGE gel. Immunoblotting was detected with anti-H3 (HuaBio, M1309-1), anti-H3K36me2 (Abcam, ab9049), anti-H3K36me3 (Abcam, ab9050) and anti-H3K27me3 (Abcam, ab6002) respectively. The relative intensity of western blots was quantified with ImageJ software.

### mRNA expression analysis

Fresh mycelia cultured in a liquid CM for 2 d were collected. Total RNA was extracted with NGZOL reagent (HLingene, NG303M) according to the manufacturer’s instructions. Subsequently, total RNAs were reverse-transcribed into cDNAs with commercial kits (Toyobo, FSQ-301). Real-time PCR (RT-qPCR) was performed with SYBR Green qPCR Master Mix (Toyobo, QST-100) in a LightCycler480 system (Roche). The constitutively expressed *Tubulin* (*MGG_00604*) was used as endogenous control to normalize the amount of cDNA templates. Primers used in the experiments are listed and described in Table S2.

### RNA sequencing (RNA-seq) analysis

Fresh mycelia cultured in a liquid CM for 2 d were collected. Total RNAs were extracted and sequenced using the Illumina HiSeq X-Ten system and the Hiseq-PE150 strategy by the Novogene Corp (Beijing, China). RNA-seq analysis was conducted as previously described (Lin et al. 2022). Genes with at least a two-fold change in expression levels (*P* < 0.05) were considered differentially expressed. The downstream personalized analysis was mainly analyzed and mapped with RStudio software (R version 4.1.3). PCA analysis was carried out by plotPCA using DEseq2 software. Gene Ontology (GO) analysis with enriched biological processes was performed using DAVID (https://david.ncifcrf.gov/home.jsp) with default settings.

### Chromatin immunoprecipitation (ChIP) and ChIP sequencing (ChIP-seq) analysis

ChIP experiments were conducted with mycelia as previously described (Lin et al. 2022). Briefly, 1.0 g mycelia were crosslinked with 1% formaldehyde and stopped with 125 mM glycine. After grinding and nuclei isolation, chromatin was extracted and sonicated into 200 ~ 500 bp fragments with Diagenode Bioruptor (high setting, 16 cycles with 30 s on/30 s off). The chromatin was incubated with anti-H3K27me3 (Abcam, ab6002), anti-H3K36me2 (Abcam, ab9049) or anti-H3K36me3 (Abcam, ab9050) antibodies for 8 ~ 12 h. Subsequently, DNA was purified using a method with phenol–chloroform extraction. The recovered DNAs were used as templates for subsequent ChIP-seq. Two biological repeats were conducted for each experiment.

For ChIP-seq assay, the recovered DNAs were used for library construction with the Scale ssDNA-seq Lib Prep Kit (ABclonal, RK20228). High-throughput sequencing was conducted with Illumina HiseqPE150 system by the Novogene Corp. After sequencing, clean reads were obtained with the quality control of FASTP (v. 0.20.0) and mapped to the reference genome using BOWTIE2 (v.2.3.5) software with default parameters and reads with low mapping quality or multiple positions in the genome were identified and removed using SAMTOOLS (v.1.9) (Langmead and Salzberg 2012). Enriched peaks were called and annotated by HOMER (v.4.9.1) using ‘getDifferentialPeaksReplicates.pl -style histone, -f 2, -q 0.05, -DESeq2’. DEEPTOOLS (v.3.3.0) was used for the following (Ramirez et al. 2016): (1) BAMCOVERAGE programme with ‘-bs 10’ was used to convert bigwig files, which were imported into Integrative Genomics Viewer (IGV) for visualisation. (2) BAMCOMPARE was used to normalize and obtain log2ratios from two BAM files using ‘-bs 10, --operation log2, --pseudocount 0.001, -of bigwig’. (3) COMPUTEMATRIX and PLOTHEATMAP programmes were used to create meta-plots to compare the average levels of H3K36me2, H3K36me3 and H3K27me3 at defined loci between WT and mutants (Ramirez et al. 2016). To assign peaks to proximal genes, 1.0-kb or 3.0-kb flanking the peak summit were extracted for further analysis.

### Phylogenetic analysis

Protein sequences of Ash1 (MGG_02937) and Set2 (MGG_01661) orthologues from different species were retrieved from NCBI using the Basic Local Alignment Search Tool (BLAST) as a query. The sequences were first aligned with ClustalW algorithm and phylogenetic analysis was conducted with MEGA software (v.11.0.10) (Tamura et al. 2021). Subsequently, schematic protein diagrams were drawn by SMART (http://smart.embl-heidelberg.de/).

### Statistical analysis

Two-tailed *t* test was calculated with Excel. One-way ANOVA analysis was performed with GraphPad Prism. *P* values were calculated with the Fisher’s exact test for overlapping using online tools to determine the significance of the overlap of two gene sets (the total number of genes used in the *M. oryzae* genome was 14317) (http://nemates.org/MA/progs/overlap_stats.html). Correlation analysis between H3K36me density and relative expression was calculated with Excel and Rstudio, of which cor function was used with *Pearson* method. It was normalized by log_2_(FPKM) of ChIP-seq data and normalized by log_2_(counts) of RNA-seq data.

### Supplementary Information

Below is the link to the electronic supplementary material.

Supplementary file1 (DOCX 4150 KB)

## Data Availability

The ChIP-seq and the RNA-seq datasets generated in this article were deposited in the Gene Expression Omnibus (GEO) under the accession number GSE235415, GSE235260 and GSE235261.
