# Supplementary material for: Two H3K36 methyltransferases differentially associate with transcriptional activity and enrichment of facultative heterochromatin in rice blast fungus
Source: aBIOTECH. 2023 Dec 18;5(1):1–16. doi: 10.1007/s42994-023-00127-3 (PMC10987451; doi:10.1007/s42994-023-00127-3)
Supplement: Supplementary file 1 — Supplementary file1 (DOCX 4150 KB) [file 42994_2023_127_MOESM1_ESM.docx]

**Two H3K36 methyltransferases differentially associate with transcriptional activity and enrichment of facultative heterochromatin in rice blast fungus**

**Supplementary Information**

**Fig. S1** Evolutionary analysis and homology comparison of Ash1 and Set2 among fungal pathogens.

**Fig. S2** Identification of deletion mutants of *ASH1* and *SET2*.

**Fig. S3** Relative expression of pathogenesis-related genes in the indicated strains.

**Fig. S4** Subcellular localization of Ash1-GFP and Set2-GFP in the mycelia and conidia.

**Fig. S5** Relative protein abundance of H3K36me2/3 and H3 in the indicated strains.

**Fig. S6** Overlap and genomic distribution of H3K36me2/3-marked regions in *M. oryzae*.

**Fig. S7** Ash1-established H3K36me2 and Set2-established H3K36me3 specifically associate with transcriptional repression and activation respectively.

**Fig. S8** Transcriptome analysis of DEGs in the Δ*ash1* and Δ*kmt6* stains.

**Fig. S9** Relative abundance of H3K27me3 and H3 in the indicated strains.

**Fig. S10** Gene ontology (GO) analysis.

**Fig. S11** Ash1 and Set2 differentially regulate stress-responsive genes.

**Fig. S12** Relative abundance of H3K36me2/3 and H3 in the indicated strains with (2 h) or without stress treatment (0 h).

**Table. S1** Strains used in this study.

**Table. S2** Primers used in this study.

**
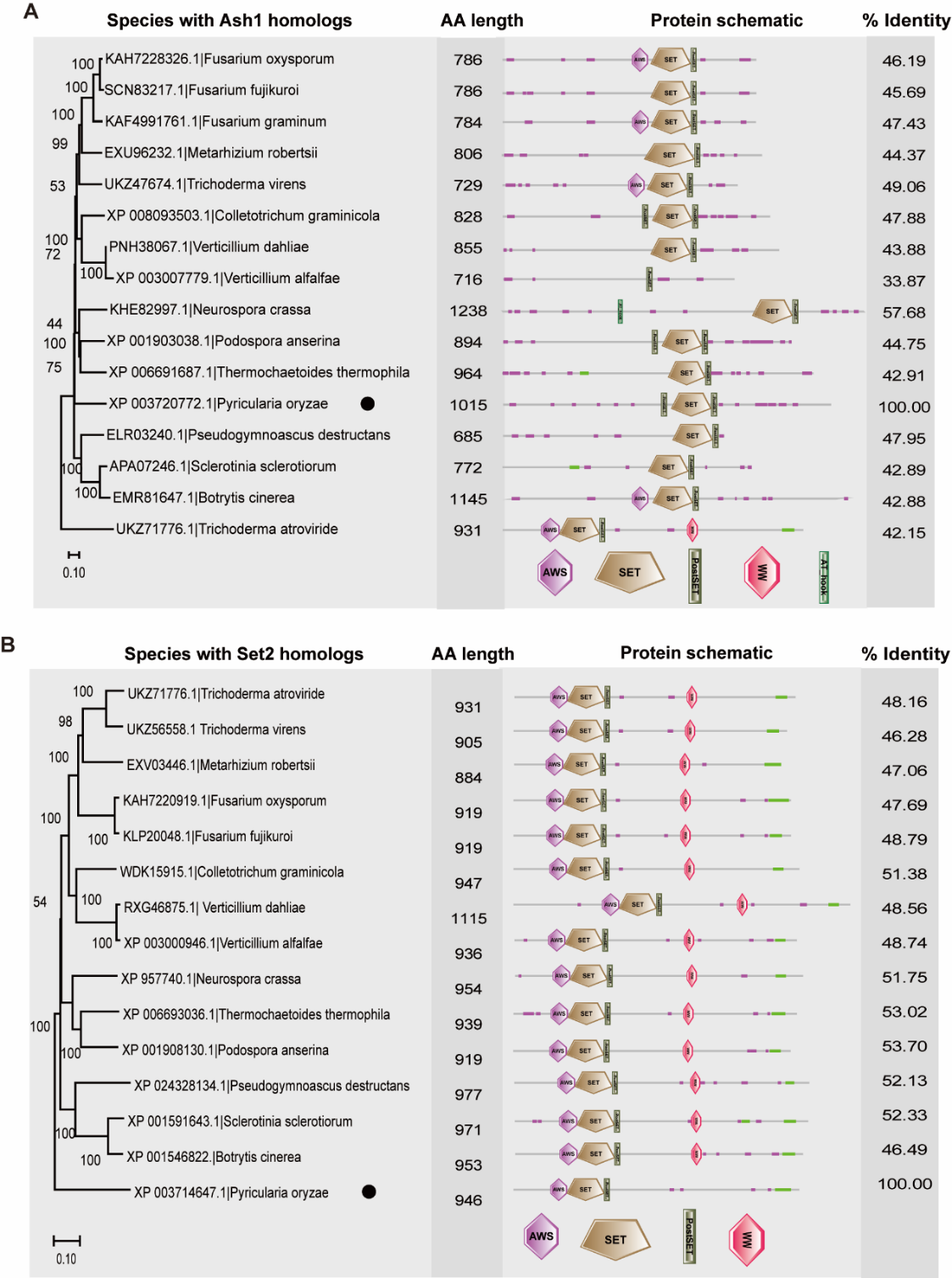
**

**Fig. S1** Evolutionary analysis and homology comparison of Ash1 and Set2 among fungal pathogens. (A-B) Phylogenetic relationships and conserved domains of Ash1 and Set2 orthologs are shown among fungi species respectively. The number of amino acids and sequence identity from different fungal species compared with Ash1 and Set2 from *M. oryzae* are shown*.* The percentage of phylogenetic tree in which the associated taxa clustered together in the bootstrap test (1000 replicates) is shown at branch nodes. The tree is drawn to scale, with branch lengths in the same units as those of the evolutionary distances used to infer the phylogenetic tree.

**
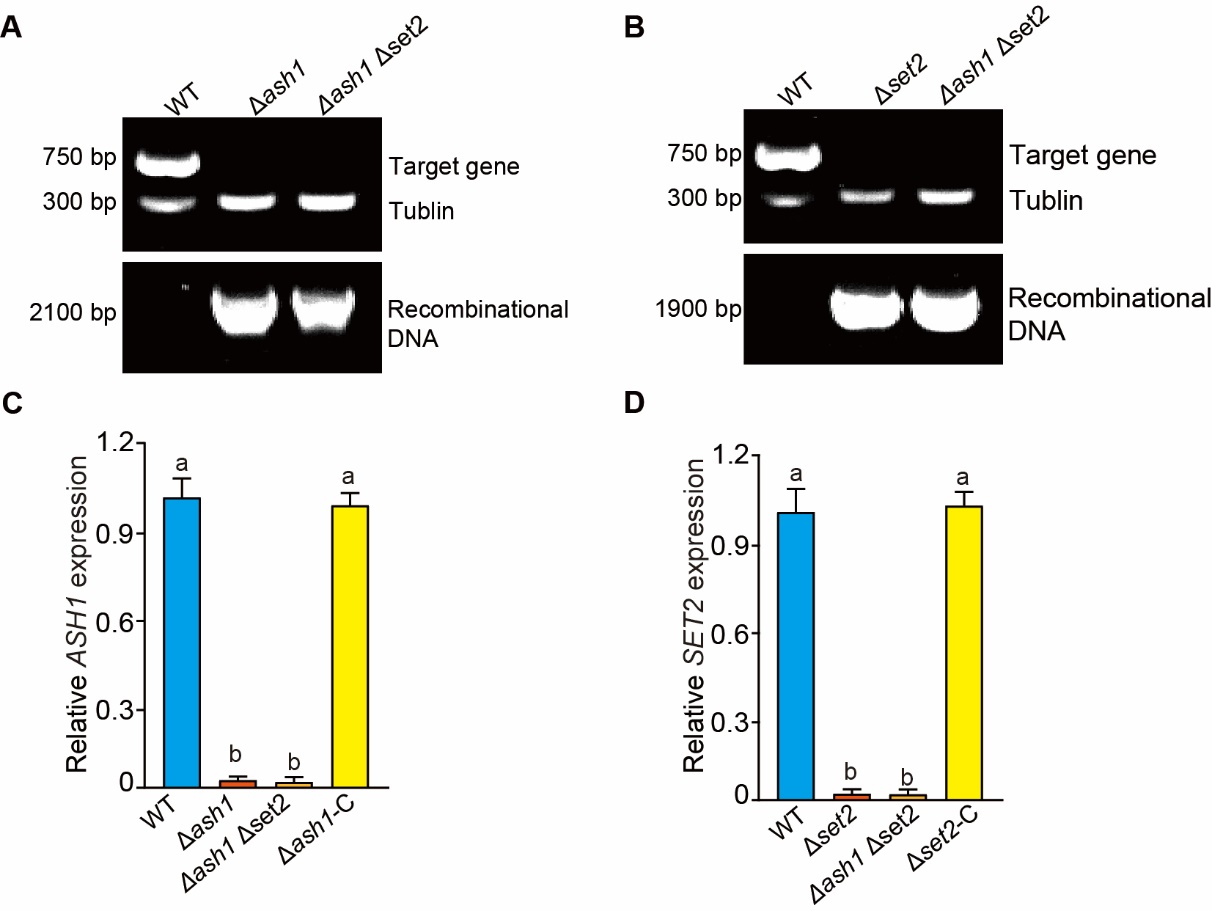
**

**Fig. S2** Identification of deletion mutants of *ASH1* and *SET2*. (A-B) PCR identification of the indicated strains. (C-D) Expression analysis of *ASH1* and *SET2* in the indicated strains respectively. Values are the means ± standard deviation from three technical repeats and different letters (a or b) indicate significant differences tested by a one-way ANOVA (*P* < 0.05).

**
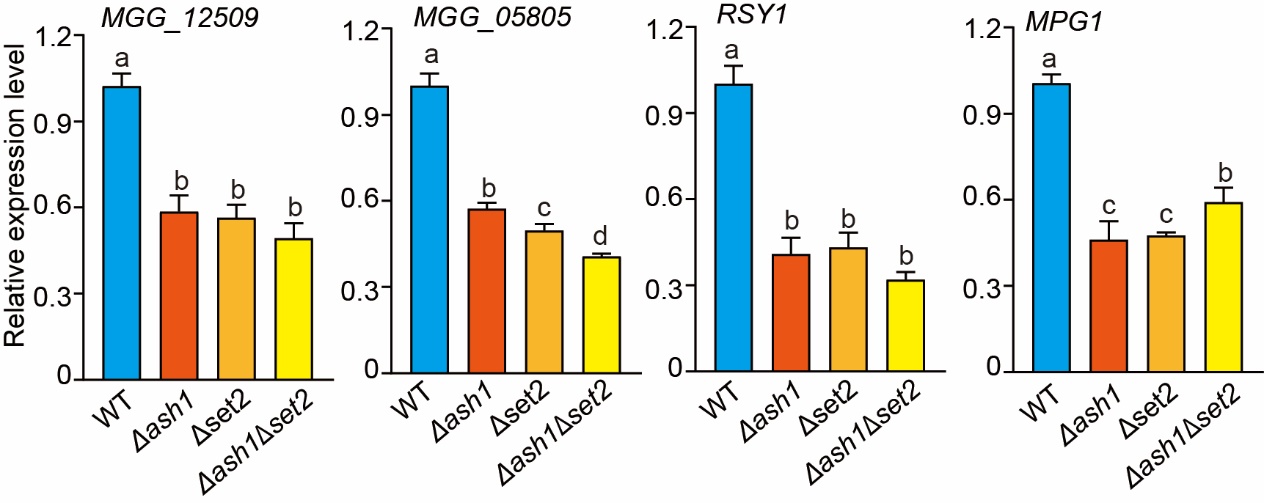
**

**Fig. S3** Relative expression of pathogenesis-related genes in the indicated strains. Values are the means ± standard deviation from three biological repeats and different letters (a or b) indicate significant differences tested by a one-way ANOVA (*P* < 0.05).

**
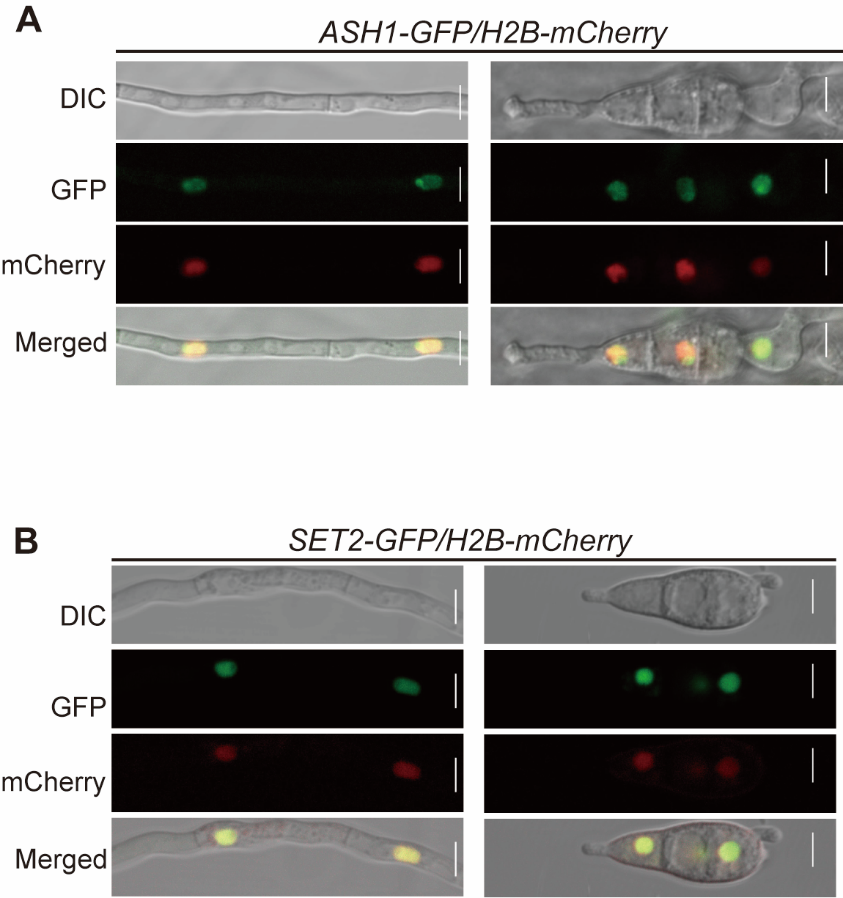
**

**Fig. S4** Subcellular localization of Ash1-GFP and Set2-GFP in the mycelia and conidia. *H2B-mCherry* was transformed and co-expressed in the *ASH1-GFP* and *SET2-GFP* strains respectively. Bar, 5 µm.

**
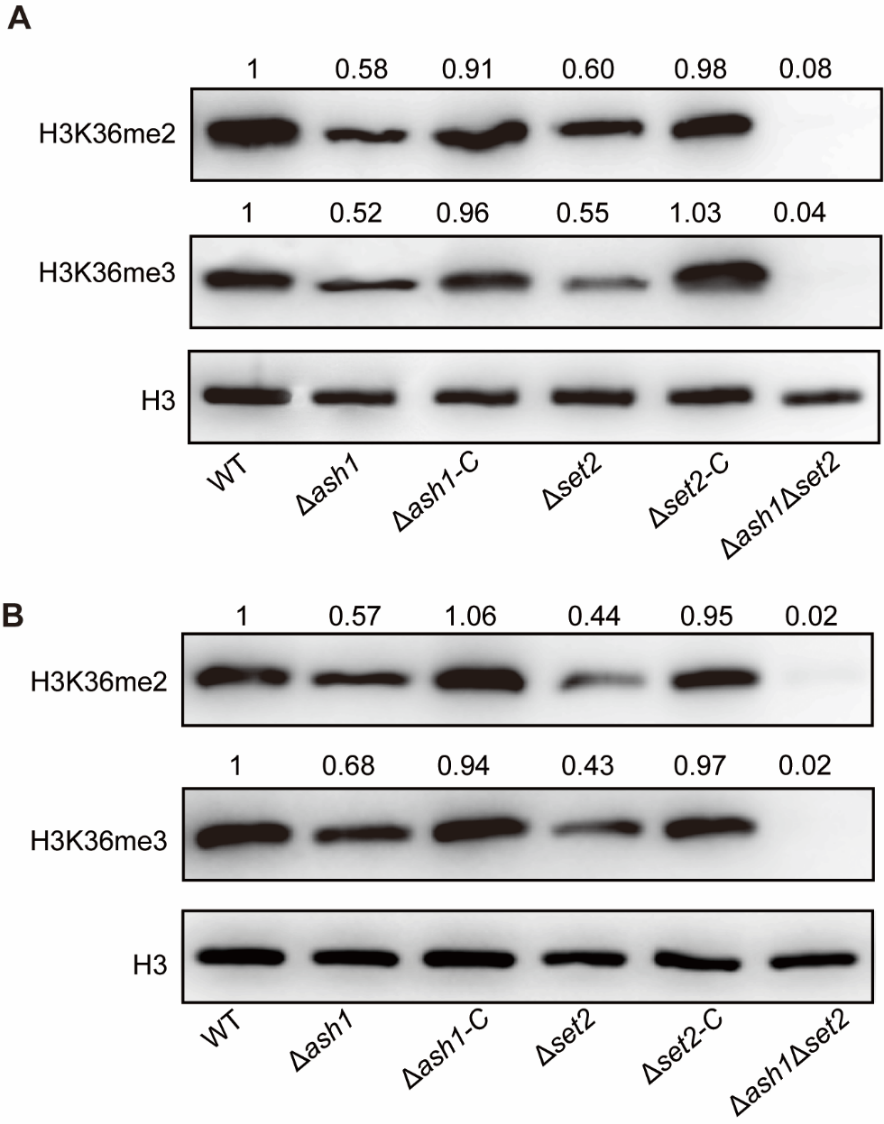
**

**Fig. S5** Relative protein abundance of H3K36me2/3 and H3 in the indicated strains. The relative abundance was measured and calculated relative to that of the WT strain with ImageJ software.

**
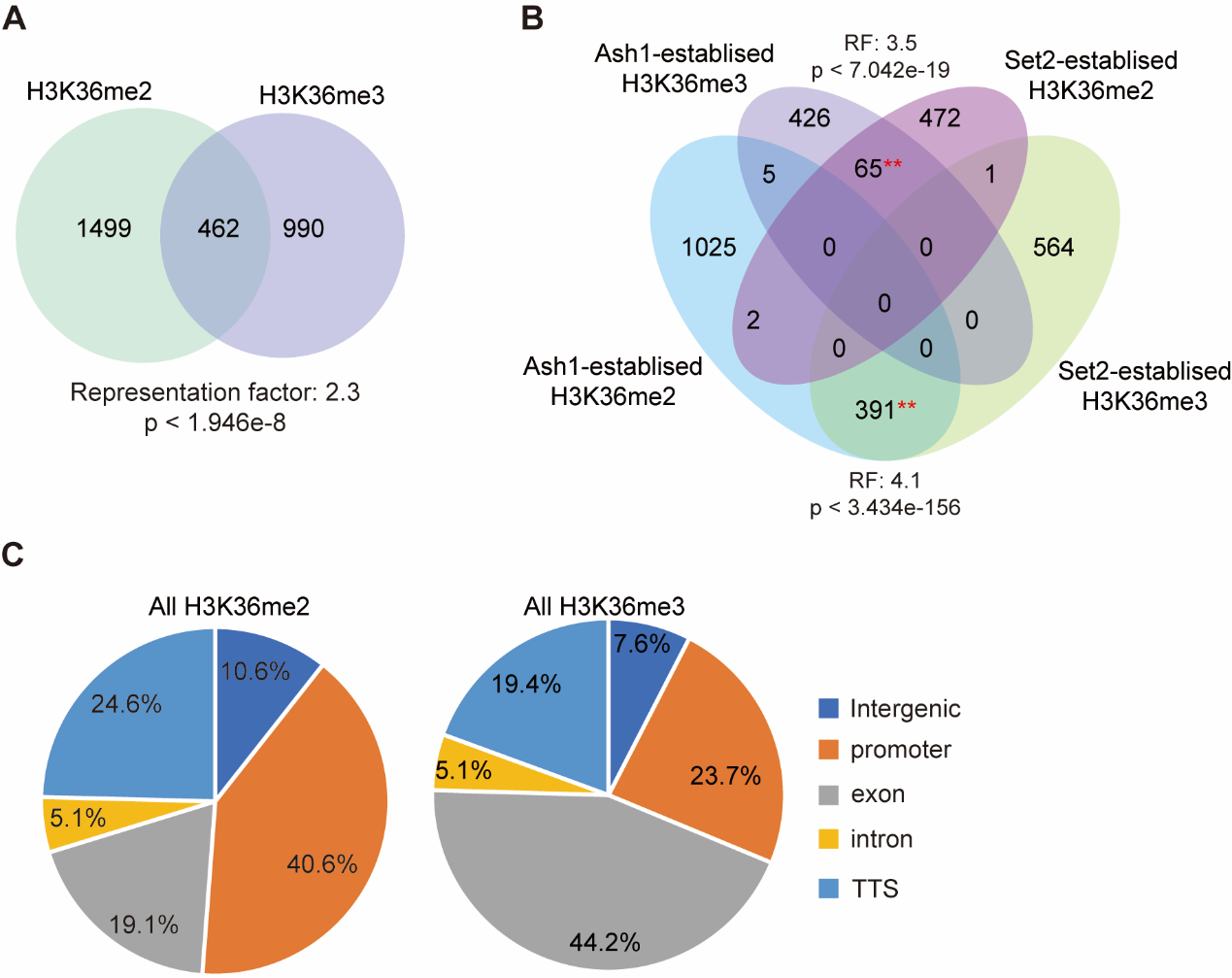
**

**Fig. S6** Overlap and genomic distribution of H3K36me2/3-marked regions in *M. oryzae*. (A) Venn diagram showing overlap of marked genes with all H3K36me2 and H3K36me3. (B) Venn diagram showing overlap of marked genes with Ash1-established H3K36me2/3 and Set2-established H3K36me2/3. ** indicates significant overlap tested between two gene sets (*P* < 0.01). (C) Genomic distributions of H3K36me2/3-marked regions are shown.

**
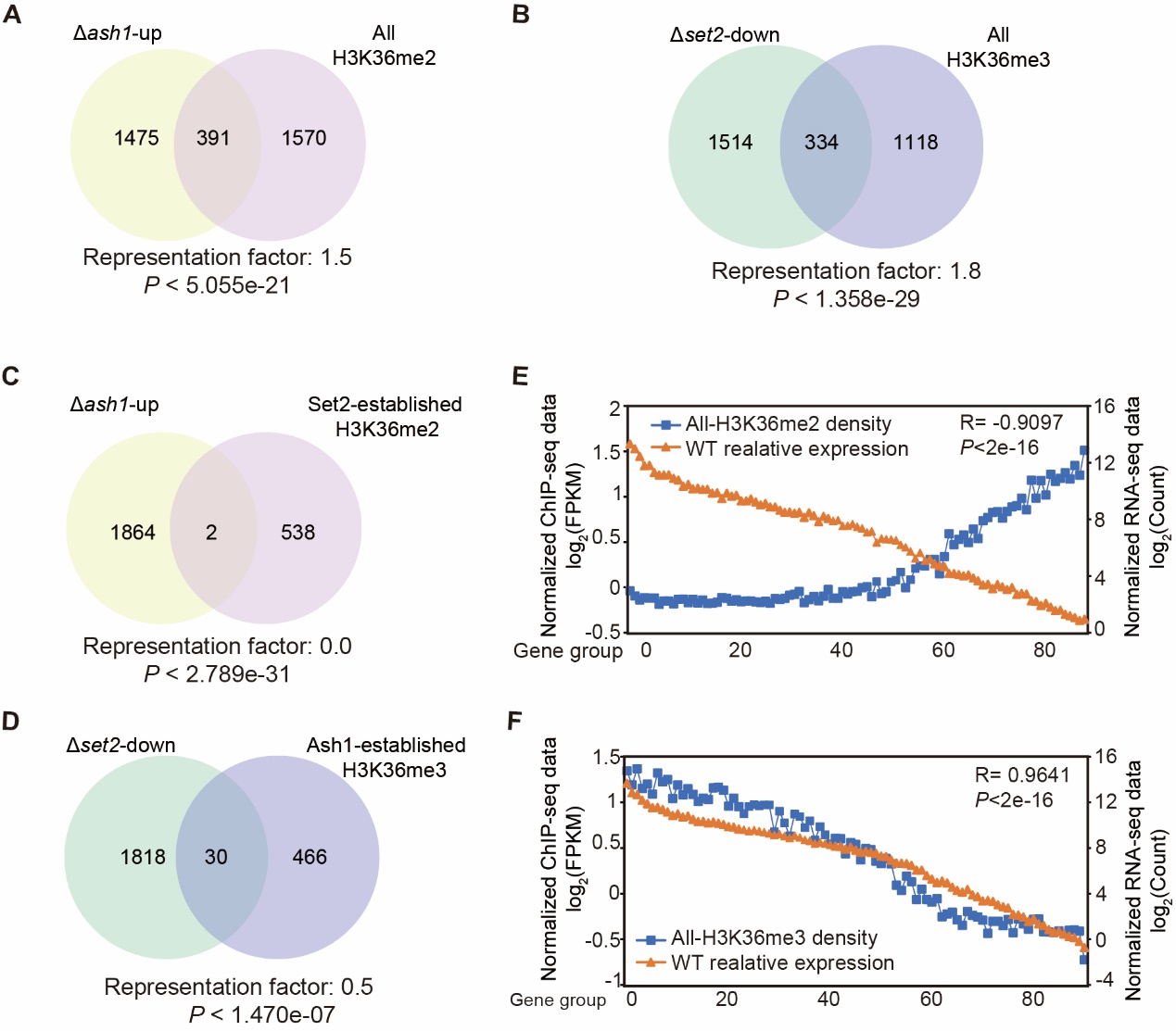
**

**Fig. S7** Ash1-established H3K36me2 and Set2-established H3K36me3 specifically associate with transcriptional repression and activation respectively. (A) Venn diagram showing significant overlap of H3K36me2-marked genes and *∆ash1*-up. (B) Venn diagram showing significant overlap of H3K36me3-marked genes and *∆set2*-down. (C) Venn diagram showing no overlap of Set2-established H3K36me2 marked genes and *∆ash1*-up. (D) Venn diagram showing overlap of Ash1-established H3K36me3 marked genes and *∆set2*-down. (E) Correlation analysis between H3K36me3 ChIP-seq and gene expression in RNA-seq. A total of 14317 genes were divided into 100 groups and sorted by descending WT relative expression level. 91 gene expression groups are shown. x-axis: gene groups; left y-axis: normalized ChIP-seq data; right y-axis: normalized RNA-seq data. (F) Correlation analysis between H3K36me2 ChIP-seq and gene expression in RNA-seq.

**
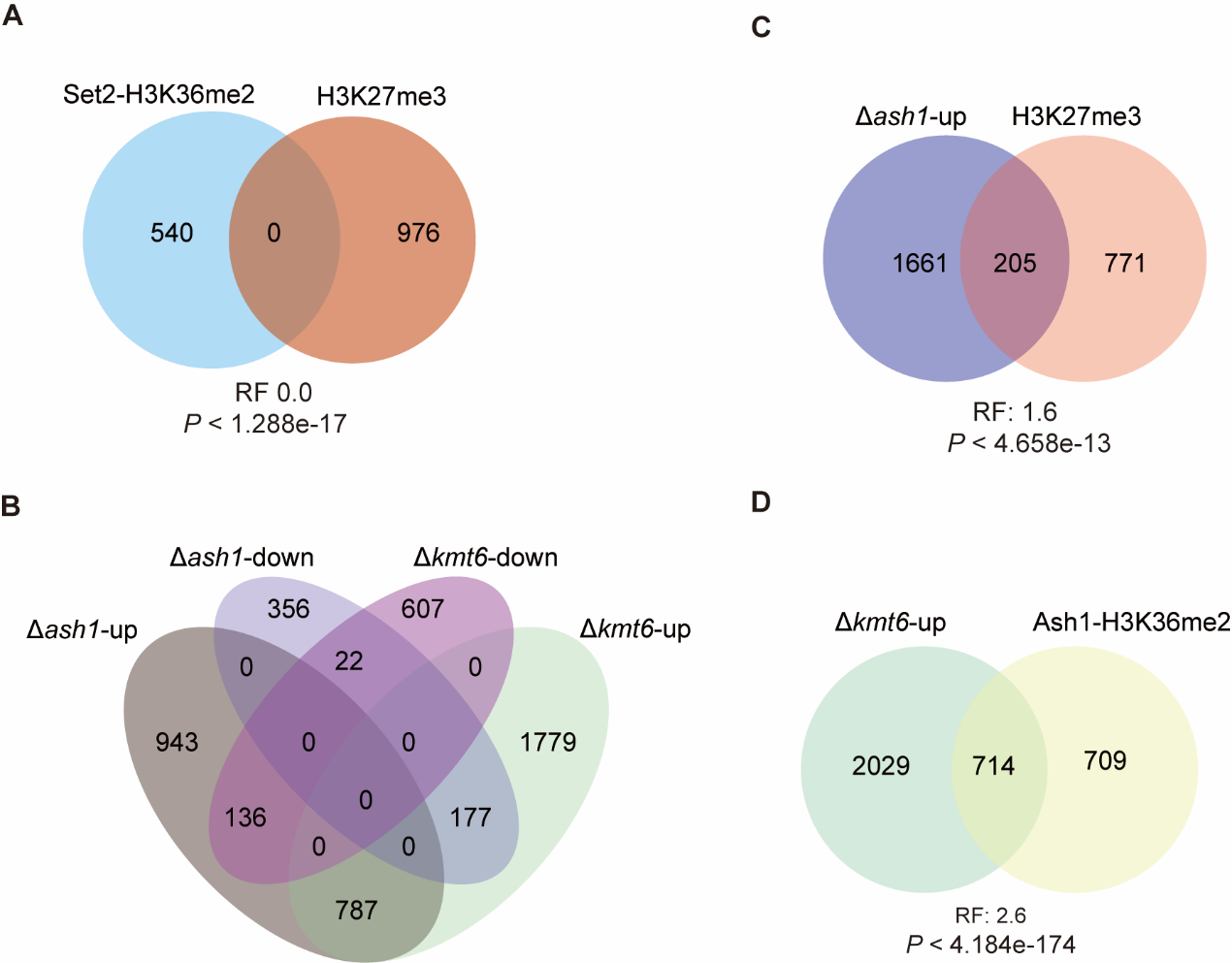
**

**Fig. S8** Transcriptome analysis of DEGs in the Δ*ash1* and Δ*kmt6* stains. (A) Venn diagram showing no overlap of Set2-established H3K36me2 and H3K27me3-marked genes. (B) Transcriptome analysis of DEGs in the Δ*ash1* and Δ*kmt6* stains. (C) Venn diagram showing overlap of H3K27me3-marked genes and *∆ash1*-up. (D) Venn diagram showing overlap of Ash1-H3K36me2-occupied genes and *∆kmt6*-up.

**
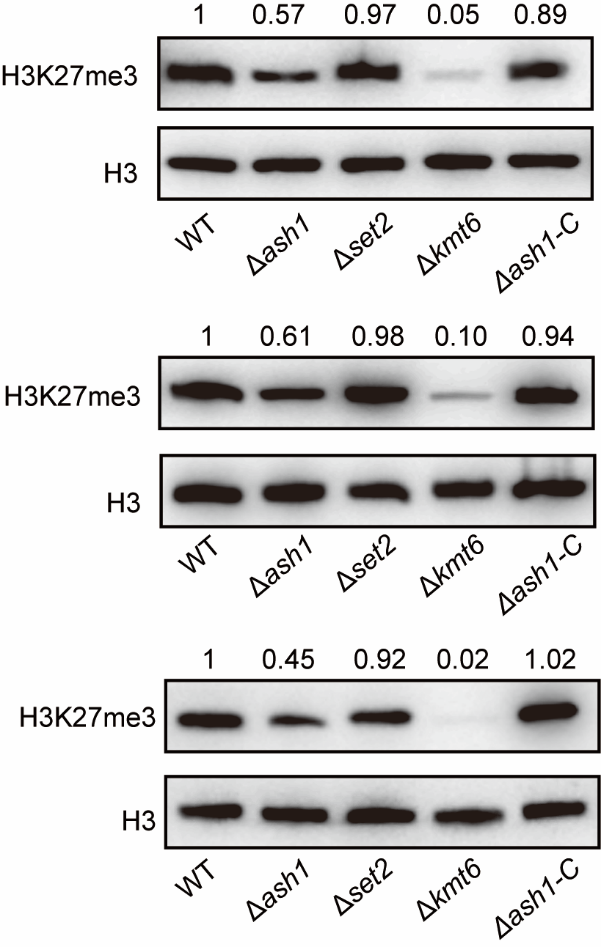
**

**Fig. S9** Relative abundance of H3K27me3 and H3 in the indicated strains. The relative abundance was measured and calculated relative to that of the WT strain with ImageJ software.

**
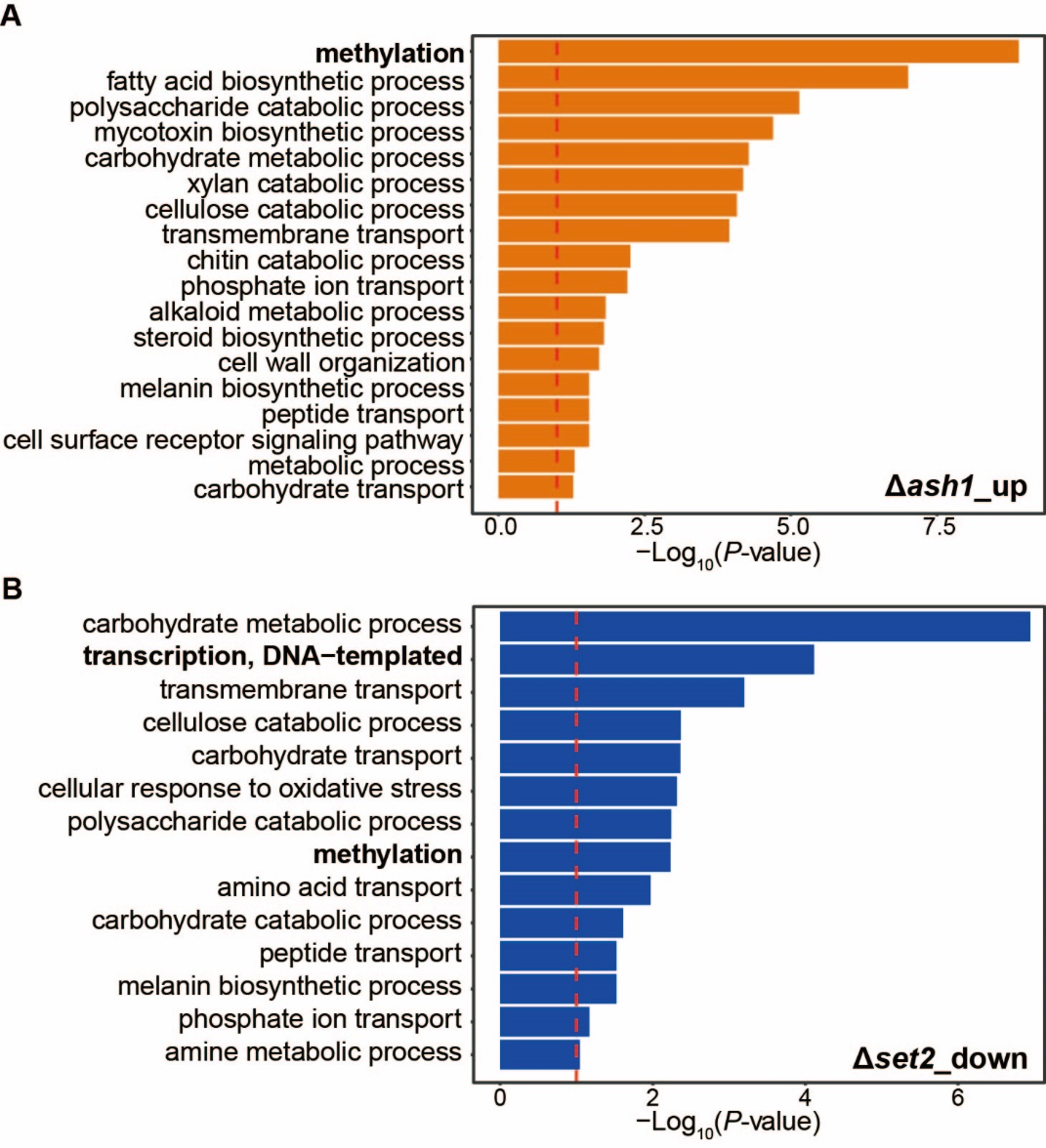
**

**Fig. S10** Gene ontology (GO) analysis. (A) GO analysis of up-regulated genes in the Δ*ash1* strain (Δ*ash1*_up). (B) GO analysis of down-regulated genes in the Δ*set2* strain (Δ*set2*_down).

**
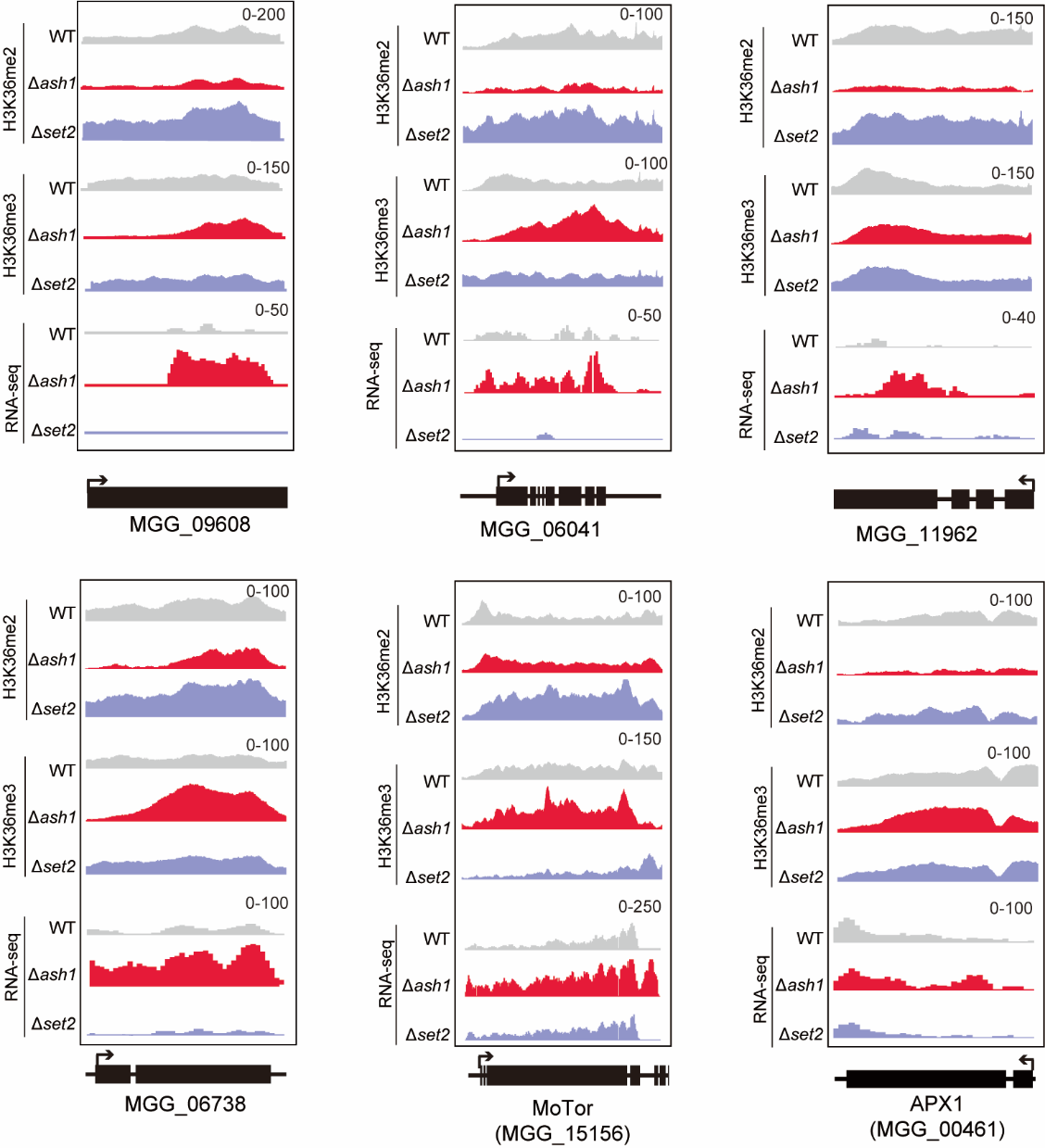
**

**Fig. S11** Ash1 and Set2 differentially regulate stress-responsive genes. Integrative Genomics Viewer (IGV) of ChIP-seq and RNA-seq in the WT, Δ*ash1* and Δ*set2* strains are shown. The number indicates reads per million (RPM).

**
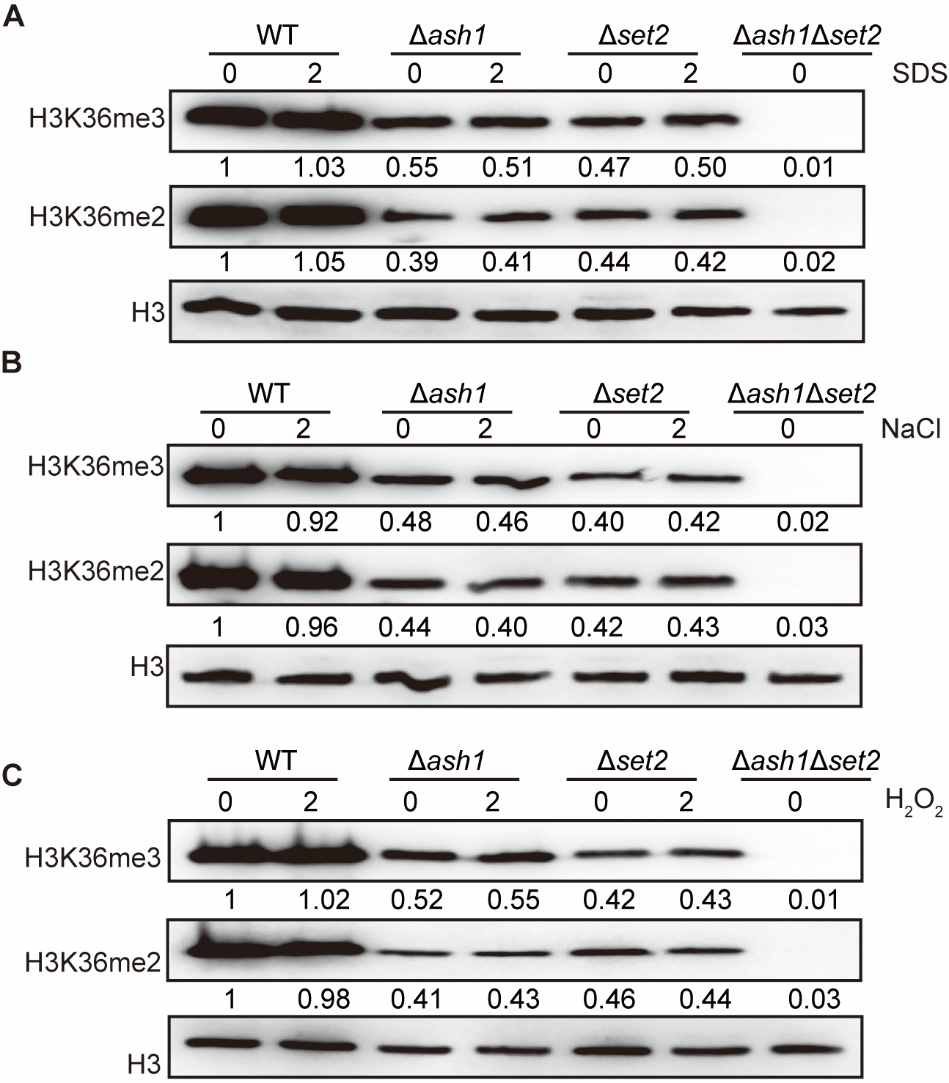
**

**Fig. S12** Relative abundance of H3K36me2/3 and H3 in the indicated strains with (2 h) or without stress treatment (0 h). The relative abundance was measured and calculated relative to that of the WT strain with ImageJ software. “0” or “2” indicated the hours after stress treatment. Ratio of H3K27me3 to H3 was calculated and the ratio in the WT strain was set “1”.

**Table. S1** Strains used in this study.

| Strain | Description |
| --- | --- |
| B157 | Wild-type |
| Δ*ash1* | deletion mutant of *MGG_02937* in B157 |
| Δ*ash1*-C | expressing *ASH1_pro_-ASH1* in Δ*ash1* transformant |
| Δ*set2* | deletion mutant of *MGG_01661* in B157 |
| Δ*set2*-C | expressing *SET2_pro_-SET2:GFP* in Δ*set2* transformant |
| Δ*ash1*Δ*set2* | deletion mutant of *MGG_01661* in Δ*ash1* transformant |
| Δ*kmt6* | deletion mutant of *MGG_00152* in B157 |
| *ASH1-GFP/H2B-mCherry* | expressing *H3_pro_-ASH1:GFP* in H2B-mCherry transformant |
| *SET2-GFP/H2B-mCherry* | expressing *H3_pro_-SET2:GFP* in H2B-mCherry transformant |

**Table. S2** Primers used in this study.

| Primer name | Sequence (5’-3’) | Usage |
| --- | --- | --- |
| Ahs1-5F | agtCTCGAGAGGTACACCAGTAGGGCACCTG | cloning *ASH1* 5' flanking sequence for deletion |
| Ash1-5R | tgaGAGCTCCGAATCAAGTTGAAATACGTCGGT |  |
| Ash1-3F | tgaGTCGACAAACCGAGTGCGAAGTCTGC | cloning *ASH1* 3' flanking sequence for deletion |
| Ash1-3R | cgtGCATGCAGGCAAGGCAAGATTGGCTAAG |  |
| Ash1-upF | ACGGCTTCGCACTTACGGGC | amplifying Δ*ash1* recombination fragments for identification of mutants |
| P821-5R | ACCTCCACTAGCTCCAGCCAAG |  |
| Tubulin-gF | CACCTGCTTGCGTTTCCC | Amplifying *β-tubulin* inner sequence for identification of mutants |
| Tubulin-gR | TACGACGAGTTCTTGTTCTG |  |
| Ash1-innerF | CATGATCTTGGATGCTACTACTGGCA | Amplifying *MGG_02937* inner sequence for identification of mutants |
| Ash1-innerR | CGTGGCCTTTGTCTTGCTGGT |  |
| Set2-5F | agtGAATTCGTTGACATCTGGTTTGAAGCAATG | cloning *SET2* 5' flanking sequence for deletion |
| Set2-5R | agtGGATCCGCACCTTCCATTCCTCGCAA |  |
| Set2-3F | agtCTGCAGAAGGGTGGGACCTGGTTCCT | cloning *SET2* 3' flanking sequence for deletion |
| Set2-3R | agtAAGCTTCGACCACTGGAACCTGTCGAG |  |
| Set2-upF | TCAGGTATTATCGCTCTTCTCCACC | amplifying Δ*set2* recombination fragments for identification of mutants |
| P822-5R | GGGCTGATCTGACCAGTTGCC |  |
| Set2-innerF | ATCCTGGGTGGCAAGACCCA | amplifying *MGG_01661* inner sequence for identification of mutants |
| Set2-innerR | CCATCCAGACGGTAGTGCAGGA |  |
| Tubulin -qF | CTGCCATCTTCCGTGGAAAGG | amplifying *β-tubulin* for RT-qPCR |
| Tubulin -qR | GACGAAGTACGACGAGTTCTTG |  |
| RT-12509-F | GCTCGCACGGCCCGGT | amplifying *MGG_12509* for RT-qPCR |
| RT-12509-R | GCGGTCTACCGTCGTCTTTGAGC |  |
| RT-05805-F | AGGAGTCCGTCGCCCACGTC | amplifying *MGG_05805* for RT-qPCR |
| RT-05805-R | TTCATGCACTGCGTGGTGCC |  |
| RT-RSY1-F | CGCATTGACTACCGCTCCTTCCT | amplifying *RSY1* for RT-qPCR |
| RT-RSY1-R | CGCGTGCCGCCGATGA |  |
| RT-MPG1-F | CAGCGGCGAGTGCAAGAACA | amplifying *MPG1* for RT-qPCR |
| RT-MPG1-R | GTTGACCAGACCAATCTGCTCGC |  |
| RT-09608-F | CGCTTCGCCACACCAGGCT | amplifying *MGG_09608* for RT-qPCR |
| RT-09608-R | CTCAATCCCAGTCCTTTCATCGGA |  |
| RT-06041-F | CCTGGATCATCCCATATTACCATTCG | amplifying *MGG_06041* for RT-qPCR |
| RT-06041-R | AGGCTCAACACGATCTTGTTCTCCC |  |
| RT-11962-F | AAGAACCTTACCGGACATGGAAGCTAC | amplifying *MGG_11962* for RT-qPCR |
| RT-11962-R | GCGGTGGCGTGATCGAGTGT |  |
| RT-06738-F | TGATTCAAATGTTCTTACCGGCGG | amplifying *MGG_06738* for RT-qPCR |
| RT-06738-R | TGAAGAGATAGGGTATCTCCATCTTGCG |  |
| RT-MoTor-F | GCTTTCATCCACGACCCGCTC | amplifying *MoTor* for RT-qPCR |
| RT-MoTor-R | GGGCTCGGGTCGCAGCA |  |
| RT-APX1-F | GGTCGCATTCGGGTTAGGACG | amplifying *APX1* for RT-qPCR |
| RT-APX1-R | CGTAAAGACCAGTGGCATTTCCAGA |  |
